# From genes to clinical application: a circulating four-gene signature for early diagnosis model of refractory *Mycoplasma pneumoniae* pneumonia

**DOI:** 10.3389/fcimb.2026.1741058

**Published:** 2026-04-07

**Authors:** Qiuting Wu, Zhiwei Lu, Xuehui He, Heping Wang, Xiaoli Zhang, Xiangtao Wu, Weihong Lu, Qi Feng, Qiru Su, Xingliang Zhang

**Affiliations:** 1 Department of Respiratory Medicine, Affiliated Shenzhen Children’s Hospital of Shantou University Medical College, Shenzhen, China; 2 Institute of Pediatrics, Shenzhen Children’s Hospital, Shenzhen, China; 3 Department of Laboratory Medicine, Laboratory Medical Immunology, Radboud University Medical Center, Nijmegen, Netherlands; 4 Department of Pediatrics, First Affiliated Hospital of Xinxiang Medical University, Weihui, China; 5 Department of General Surgery, Shenzhen Children’s Hospital, Shenzhen, China; 6 Clinical Research Academy, Peking University Shenzhen Hospital, Shenzhen, China; 7 Clinical Research Department, Clinical Laboratory, Affiliated Shenzhen Children’s Hospital of Shantou University Medical College, Shenzhen, China

**Keywords:** biomarkers, diagnostic model, GMPP, RMPP, single-cell RNA sequencing, three-category logistic regression

## Abstract

**Objective:**

Refractory *Mycoplasma pneumoniae* pneumonia (RMPP) presents a major clinical challenge in children, largely due to the absence of reliable early diagnostic markers, which contributes to delayed intervention and an increased risk of severe complications. This study aimed to identify early diagnostic biomarkers based on peripheral blood mononuclear cell (PBMC) gene expression profiles and to develop and validate a model capable of distinguishing RMPP from general MPP (GMPP) and healthy controls (Normal).

**Methods:**

A total of 349 children (117 Normal, 123 GMPP, and 109 RMPP) were chronologically divided into a prospective training cohort (n=295) for model development and a prospective validation cohort (n=54) for external validation. Single-cell RNA sequencing (scRNA-seq) was performed on PBMCs from a discovery cohort (n=8) randomly selected from the training cohort. Differentially expressed genes that were specifically and significantly upregulated in RMPP groups were screened as candidate early diagnostic biomarkers. After primer validation, expressions of these candidate genes were subsequently measured using RT-qPCR in the entire study population. A multinomial logistic regression model with backward selection was developed on the training set, externally validated in the validation set, and its internal validation was further assessed via 1000 bootstrap resamples of the full dataset.

**Result:**

scRNA-seq identified eight specifically upregulated genes in the RMPP group. Subsequent RT-qPCR validation in the training cohort confirmed four genes—*IGHM*, *NEAT1*, *IL32*, and *ACTG1*—as early diagnostic biomarker capable of differentiating among the three groups. A combined four-gene three-category logistic regression model (Normal/GMPP/RMPP) demonstrated strong performance, with macro-average area under the curve values of 0.968 and 0.987 in the training and external validation, respectively. The final model, refit on the full dataset, attained an overall diagnostic accuracy of 88.8% for three-category classification, which was further confirmed by bootstrap resampling (macro-average AUC = 0.969).

**Conclusion:**

We established a robust PBMC-based four-gene signature diagnostic model that accurately discriminates among Normal, GMPP, and RMPP statuses at an early disease stage. This model provides a clinically accessible and precise tool to facilitate early intervention and improve patient management.

## 1 Introduction

*Mycoplasma pneumoniae* (MP) is a major pathogen responsible for community-acquired pneumonia (CAP) in children. Pneumonia caused by MP infection, termed *mycoplasma pneumoniae* pneumonia (MPP), accounts for approximately 10%-40% of pediatric CAP cases (Sun et al., 2025). MPP predominantly affects preschool and school-aged children, is primarily transmitted via respiratory droplets, and typically follows a self-limiting course with a favorable prognosis in most cases (Xing et al., 2020). However, a subset of children does not respond adequately to conventional macrolide therapy and may progress to refractory MPP (RMPP). The early identification of RMPP is challenging. It progresses rapidly, responds poorly to standard macrolide antibiotics, and is prone to develop severe pulmonary and extrapulmonary complications (e.g., necrotizing pneumonia, pulmonary embolism, bronchial mucus plugs), potentially leading to death (Xu et al., 2024). This significantly increases the healthcare burden, patient suffering, and family psychological distress. Notably, the resistance rate of MP to macrolides in China has been persistently rising (Li et al., 2024; Xing et al., 2024), implying that an increasing number of children with MPP might not respond to first-line treatment, consequently developing RMPP. Therefore, the early recognition of RMPP and timely adjustment of treatment strategies are critically important for halting disease progression, reducing the risks of disability and mortality, and improving patient outcomes.

There is currently no clear, universally accepted diagnostic criterion for RMPP in children. The definition proposed by Japanese researchers in 2008 is widely referenced, characterizing RMPP as persistent fever and worsening radiographic findings after at least 7 days of appropriate macrolide antibiotic therapy (Tamura et al., 2008). Consistent with this, the latest Chinese CAP guidelines suggest considering RMPP in children with MPP who, after 7 days or more of standard macrolide treatment, exhibit persistent fever, worsening clinical signs and chest imaging, or the development of extrapulmonary complications (Subspecialty Group of Respiratory et al., 2025). The early diagnosis of RMPP remains a clinical challenge (Shan et al., 2017), hampered by significant limitations of existing diagnostic methods. Firstly, MP detection is fundamental for MPP diagnosis and a prerequisite for identifying RMPP. Conventional laboratory tests (e.g., complete blood count, lactate dehydrogenase (LDH), D-dimer, interleukin-6 (IL6)) and imaging primarily assess the state of infection and inflammatory severity but lack specificity for MPP or RMPP. While these parameters correlate with disease severity, their specificity is insufficient, and definitive diagnostic cut-off values are lacking (Jiang et al., 2025; Wei et al., 2024). MP-specific detection methods also have drawbacks: MP culture is time-consuming; serological tests (MP-IgG/IgM) can be negative during the early window period; and real-time quantitative polymerase chain reaction (RT-qPCR) for MP-DNA or RNA, while highly sensitive, offers limited value in predicting progression to RMPP. Furthermore, the predominant RT-qPCR method relies on throat swab sampling, which is often poorly tolerated and yields suboptimal sample quality in the primary MPP-affected population (infants and preschoolers) due to low cooperation (Tong et al., 2022). Regarding drug resistance, although approximately 18.67% of RMPP cases occur in patients infected with macrolide-sensitive strains, and over 20 mutations in the 23S rRNA domain and ribosomal protein genes have been linked to resistance (Pereyre et al., 2007), commercial RT-qPCR kits typically target only the A2063G/A2064G mutations, potentially missing other clinically significant resistance markers. Thus, a positive resistance test result alone is insufficient to diagnose RMPP. Researchers have continuously explored potential predictive factors through retrospective analyses, including serological markers (e.g., C-reactive protein, LDH, D-dimer, neutrophil percentage) and clinical/imaging characteristics (e.g., fever, pulmonary consolidation), aiming to build predictive models (Suber et al., 2018; Silvin et al., 2020; Lai et al., 2010). However, these studies inevitably possess methodological limitations.

Beyond these conventional approaches, host gene expression-based diagnostics have emerged as clinically validated strategies for infectious diseases. For instance, SeptiCyte LAB, a US Food and Drug Administration-cleared molecular test, quantitatively assesses the probability of sepsis by measuring the expression levels of four specific genes (*CEACAM4*, *LAMP1*, *PLAC8*, *PLA2G7*) in whole blood, assisting clinicians in distinguishing sepsis from non-infectious systemic inflammatory response syndrome (Miller et al., 2018). Similarly, a seven-gene set (bacterial/viral metascore) integrated with the Sepsis MetaScore has been developed to form an Integrated Antibiotics Decision Model, which accurately differentiates bacterial infections, viral infections, and non-infectious inflammation for guiding antibiotic use (Sweeney et al., 2016). More recently, metagenomic and metatranscriptomic sequencing of respiratory samples have enabled simultaneous profiling of host immune responses and the respiratory microbiome, facilitating accurate differentiation between infectious and non-infectious lower respiratory tract diseases and offering high sensitivity even when conventional microbiological tests yield negative results (Mick et al., 2023; Zou et al., 2025). These advances underscore the potential of integrating host gene expression signatures into diagnostic algorithms for infectious diseases. In this context, transcriptomic approaches using whole blood samples, such as microarray analyses (Wallihan et al., 2018) or RNA-seq (Viz-Lasheras et al., 2025a), offer the potential to assess disease severity and facilitate the differential diagnosis of MPP, providing a high-throughput and systematic perspective beyond conventional biomarkers. In summary, the current clinical differentiation between RMPP, general MPP (GMPP), and non-MPP infections primarily depends on clinical symptoms, imaging, and MP detection, which suffer from inadequate sensitivity and specificity. There is a notable lack of a combined diagnostic model capable of effectively distinguishing the three-category outcome of “healthy controls (Normal)/GMPP/RMPP”, increasing the risk of missed or misdiagnosis. Faced with the insufficient efficacy of existing clinical tests, research remains focused on homogeneous retrospective case analyses. Consequently, there is an urgent need to leverage novel technologies with high resolution and precise analytical power to explore early diagnostic biomarkers for RMPP, enabling more precise clinical stratification and intervention.

Recent advances in single-cell sequencing technologies, particularly single-cell RNA sequencing (scRNA-seq) – recognized by *Science* as the top scientific breakthrough of 2018 – have revolutionized the discovery of novel biomarkers for disease diagnosis and prognosis (Suber et al., 2018). Notably, scRNA-seq analysis of COVID-19 patients revealed the disappearance of non-classical CD14^Low^CD16^High^ monocytes, an increase in HLA-DR^Low^ classical monocytes, and a massive release of calprotectin (S100A8/S100A9) in severe cases, suggesting that plasma calprotectin levels and decreased abundance of non-classical monocytes could serve as discriminative markers for disease severity (Silvin et al., 2020). Growing evidence indicates that the pathogenesis of RMPP is closely associated with the host immune response, where a moderate response facilitates MP clearance (Lai et al., 2010), while excessive inflammation may exacerbate clinical symptoms and promote RMPP progression. Importantly, RMPP-associated lung injury appears to be mediated primarily by aberrant host immunity rather than direct pathogen damage (Waites and Talkington, 2004; Tanaka et al., 2002). We therefore hypothesize that distinct immune cell responses – in both pulmonary tissues and peripheral blood – may underlie the differential progression between RMPP and GMPP patients. Systematic characterization of these immunological differences could yield clinically actionable biomarkers for the early diagnosis of RMPP. Despite this potential, no scRNA-seq studies have been reported for any specimen types (pediatric or adult) comparing RMPP and GMPP patients.

In this study, we employed scRNA seq to identify early diagnostic markers for RMPP. The work aims to develop the first molecular based tool for early RMPP diagnosis in children that integrates high accuracy, low cost, and clinical feasibility. Ultimately, this approach seeks to improve pediatric health outcomes, decrease the incidence of severe disease, and alleviate the associated healthcare burden.

## 2 Materials and methods

### 2.1 Research participants

This study prospectively collects clinical data and peripheral blood samples from children with MPP who are admitted to Shenzhen Children’s Hospital (the only tertiary Class A pediatric specialist hospital in Southern China) between July 2024 and July 2025. The MPP cases includes RMPP and GMPP. The collected clinical data encompass demographic characteristics (age, gender) and laboratory parameters within 24 hours of admission (white blood cell count, pulmonary imaging, and pharyngeal swab MP-DNA or MP-RNA load, etc.). Concurrently, the Normal group is established. Children in this group presented no symptoms of acute respiratory infection and were recruited from the following sources: (1) those undergoing circumcision surgery in the Department of Urology; (2) those receiving routine health examinations in the Child Health Care Department; (3) those with non-infectious, non-inflammatory neurological conditions such as tic disorders; (4) those undergoing surgery for muscular torticollis in the Department of Orthopedics; and (5) those undergoing surgery for torticollis and strabismus. Their peripheral blood samples and clinical data, including age, gender, complete blood count, and disease history, are collected.

#### 2.1.1 Inclusion criteria for the MPP group

(1) 1 year < Age < 14 years; (2) Primary clinical manifestations of fever and cough, potentially accompanied by headache, rhinorrhea, sore throat, earache, etc.; (3) Radiological confirmation of pneumonia, evidenced by patchy infiltrates, lobar consolidation, atelectasis, pleural effusion, etc.; (4) Fulfillment of at least one of the following etiological diagnostic criteria for MP: (a) A fourfold or greater increase in serum MP antibody titer during convalescence compared to the acute phase; (b) Positive MP culture; (c) Positive MP-DNA or MP-RNA test; (5) Informed consent obtained from the child’s guardians.

#### 2.1.2 Exclusion criteria for the MPP group

(1) Incomplete clinical data; (2) Underlying conditions such as chronic lung diseases (e.g., bronchopulmonary dysplasia, congenital lung malformations), bronchial foreign body, congenital heart disease, or autoimmune deficiencies; (3) Co-infection with other pathogens.

Based on our recently published studies (Lu et al., 2024; Wu et al., 2025), in addition to meeting the aforementioned MPP diagnostic criteria, confirmation of RMPP requires fulfilling all the following conditions: (1) Clinical presentation including high fever (body temperature > 38.5 °C), paroxysmal spasmodic dry cough, dyspnea, pulmonary moist rales, and radiological pulmonary infiltrates; (2) Persistence of high fever and progression of pulmonary lesions on imaging after one week of standardized azithromycin therapy. On the contrary, children who met the MPP diagnostic criteria but did not fulfill the criteria for RMPP were enrolled into the GMPP group.

#### 2.1.3 Inclusion criteria for the normal group

(1) Pediatric patients undergoing circumcision surgery, pediatric patients with strabismus, with tic disorders, or with lower limb length discrepancy, and pediatric patients scheduled for nevus (mole) removal; (2) Absence of infection as indicated by routine blood test results; (2) 1 year < Age < 14 years; (3) Absence of other respiratory system diseases; (4) Informed consent obtained from the child’s guardians.

#### 2.1.4 Exclusion criteria for the normal group

(1) Subjects who had comorbid conditions such as immune system disorders, congenital heart disease, rhinitis, sinusitis, gastroesophageal reflux disease, or other illnesses were excluded; (2) Peripheral blood sample volume less than 1 ml; (3) Total RNA quality or concentration inadequate for RT-qPCR.

Sample size estimation was performed to ensure that the combined gene model under a three-category classification (Normal/GMPP/RMPP) achieves an area under the curve (AUC) of at least 0.85, with a 95% confidence interval width no greater than 0.10. A two-sided significance level (α) of 0.05 and a statistical power (1–β) of 0.90 were applied. Based on preliminary experimental results, the minimum expected AUC for each category (Normal, GMPP, and RMPP) was set at 0.85. The allocation ratio among the three groups was 1:1:1, and the margin of error δ (half-width of the confidence interval) was defined as 0.06. Using a multiclass receiver operating characteristic curve (ROC) formula and the “ROC – Confidence Intervals for Area Under ROC Curve” module in PASS 15 software, the required sample size was calculated separately for each category. The analysis indicated that 97 subjects per group were needed, resulting in a total of 291 subjects across all three groups.

### 2.2 Peripheral blood collection and PBMC isolation

Peripheral blood samples for single-cell RNA sequencing were collected within 24 hours of admission, with approximately 2 mL of whole blood drawn and processed promptly for PBMC isolation to best reflect the early disease state of GMPP or RMPP. Specifically, whole blood was diluted 1:1 with 1× DPBS, layered over Ficoll-Paque Plus, and centrifuged at 500 ×g for 20 minutes at room temperature. The PBMC-containing buffy coat was carefully separated and washed twice with DPBS, followed by red blood cell lysis on ice. After centrifugation, the cell pellet was resuspended in RPMI/10% FBS medium containing 10% DMSO, and 1 mL aliquots were stored at −80 °C overnight before being transferred to liquid nitrogen for long-term preservation. Patients with MP pneumonia were confirmed and classified into GMPP or RMPP groups by a senior associate or chief physician in the respiratory department, based on MP-DNA testing and subsequent clinical progression. Then, the stored samples with confirmed group identity were used for the subsequent experiments. The peripheral blood samples from the Normal group were processed and stored using the same protocol as the GMPP and RMPP groups. Finally, three randomly selected samples from each group were transported to the sequencing company under specified storage and transportation requirements for scRNA-seq.

### 2.3 Library construction and scRNA-seq data processing

Upon receipt of the samples, the scRNA-seq company (Singleron Biotechnologies Co., Ltd.) performed cell resuscitation, discarded the supernatant, and resuspended the PBMCs in phosphate-buffered saline (PBS, HyClone) to obtain a single-cell suspension (>2×10^5^ cells/mL). Cell viability was assessed microscopically using trypan blue staining. Once cell viability and count met the requirements, the samples were loaded onto a microchip using the Singleron Matrix^®^ single-cell processing system. Barcoding beads were subsequently collected from the microchip. Captured mRNA on the beads was reverse-transcribed to obtain cDNA, which was then amplified via PCR. The amplified cDNA was fragmented, ligated with sequencing adapters, and used to construct scRNA-seq libraries according to the manufacturer’s instructions of the GEXSCOPE^®^ Single Cell RNA Library Kit (Singleron). Individual libraries were diluted to 4 nM, pooled, and subjected to paired-end 150 bp sequencing on an Illumina NovaSeq 6000 sequencer.

Raw single-cell RNA sequencing reads were processed using the CeleScope pipeline (https://github.com/singleron-RD/CeleScope) v1.9.0 to generate a gene expression matrix. The workflow consisted of the following steps: (1) Data preprocessing: Low-quality reads, along with poly-A tails and adapter sequences, were trimmed using Cutadapt v1.17. (2) Information extraction: Cell barcodes and unique molecular identifiers (UMIs) were parsed. (3) Sequence alignment: Reads were aligned to the GRCh38 reference genome (Ensembl release 92 annotations) using STAR v2.6.1a. (4) Quantification: UMI counts and gene expression levels per cell were quantified using featureCounts v2.0.1, resulting in an expression matrix file for subsequent analysis.

Cell quality control was performed by excluding cells with fewer than 200 detected genes, cells whose gene counts or UMI counts fell within the top 2%, and cells with mitochondrial gene content exceeding 20%. The retained cells after filtering were used for downstream analysis, yielding the average number of genes and UMIs detected per cell. Dimensionality reduction and clustering analysis were performed using Seurat v3.1.2. Gene expression was normalized and scaled using the NormalizeData and ScaleData functions, respectively. The top 2000 highly variable genes were selected using FindVariableFeatures for principal component analysis (PCA). Based on the top 20 principal components, cell clustering was performed using the FindClusters function. The Harmony algorithm was applied to mitigate batch effects between samples. Finally, cell distribution was visualized in two-dimensional space using the Uniform Manifold Approximation and Projection (UMAP) algorithm.

### 2.4 Differentially analysis and cell-type annotation

To identify differentially expressed genes (DEGs), we utilized the FindMarkers function from the Seurat package, based on the Wilcoxon rank-sum test (using default parameters). Genes meeting the following criteria were defined as DEGs: expressed in more than 10% of cells within a given cluster, with an average log fold-change greater than 0.5.

Cell type identities for each cluster were determined by integrating classical marker genes identified among the DEGs with reference to the SynEcoSys database. Expression patterns of marker genes used for cell type identification were visualized using dot plots generated via the DoHeatmap, DotPlot, and VlnPlot functions in Seurat v3.1.2. Doublet cells, defined as cells co-expressing markers of distinct cell types, were manually identified and excluded from the analysis.

### 2.5 GO term enrichment analysis

Based on our published procedures (Wang et al., 2022) for GO term enrichment analysis, which was performed using the packages “clusterProfiler” and “org.Hs.eg.db”. In detail, the gene symbol was transformed into Entrez id using the function”select”. The list of entrez id was used for GO term enrichment using the function “enrichGO” by setting the parameter as “BP” to find out all terms about biological process. We used the function “simplify” with default parameters to remove redundant terms with too many shared genes.

### 2.6 RT-qPCR

Total RNA was extracted from PBMC pellets stored at -80 °C using 1 mL of RNAiso Plus. The integrity and purity of the isolated RNA were verified by electrophoretic and spectroscopic analysis. Reverse transcription of 500 ng total RNA was carried out using the TaKaRa PrimeScript™ RT Master Mix (Perfect Real Time) kit (Takara, RR036A), strictly following the manufacturer’s instructions. RT-qPCR was performed on a LightCycler^®^ 480 System using the TaKaRa TB Green^®^ Premix Ex Taq™ II (Tli RNaseH Plus) kit (Takara, RR820A). GAPDH mRNA levels were used as an internal control for normalization. Each sample was run in technical triplicates. Relative gene expression was calculated using the 2^–ΔΔCt^ method. The experiments for all samples were conducted under blinded conditions. The sequences of all primers were provided in **Table 1**.

**Table 1:** Primers for RT-qPCR.

Gene name	Primer type	Primer sequence(5´-3´)
*IGLC2*	Forward	GGTCTCCACTCCCGCCTTGA
	Reverse	CACTCTGTTCCCGCCCTCCT
*IGHM*	Forward	AGCTGTGAAAACCCACACCA
	Reverse	AGATGGTCTGCTTCAGTGGC
*IL-32*	Forward	CTGAAGGCCCGAATGCACCA
	Reverse	CCTCATAATAAGCCGCCACTGTCT
*HSP90B1*	Forward	GTCCTAGAGTGTTTCCTCTTGGGT
	Reverse	GCCAGTTTGGTGTCGGTTTC
*ACTG1*	Forward	GTCCCAGTTGGTGACGATGC
	Reverse	CGAGCCGTGTTTCCTTCCAT
*SUB1*	Forward	CAATTAGCACTTTGCCTTTA
	Reverse	GCCCTGTCATCTTCTAAACA
*NEAT1*	Forward	CTCTTCCTCCACCATTACCAACAATAC
	Reverse	CTTCCTCCCTTTAACTTATCCATTCAC
*MMP8*	Forward	AGCATCTCCTCCAATACCTT
	Reverse	ACGCACTAACTTGACCTACA
*GAPDH*	Forward	TGGTGAAGACGCCAGTGGA
	Reverse	GCACCGTCAAGGCTGAGAAC

### 2.7 Construction and calibration of the three-classification diagnostic model

The development of the three-category diagnostic model followed a sequential process. First, the normality of each gene’s expression distribution across the three groups (Normal, GMPP, RMPP) in the training cohort (n=295) was assessed. As the data deviated from normality, the Kruskal-Wallis test was used to evaluate the overall differences in gene expression among the groups. Following this initial screening, we evaluated the discriminatory power of each gene individually using univariable multinomial logistic regression and calculated the corresponding area under the curve (AUC) using the “One-vs-Rest” approach.

Subsequently, all six genes were incorporated into a multivariable logistic regression model. The model was implemented using the multinom function from the nnet package (version 7.3-19) in R (version 4.3.0). Variable selection was conducted through a backward stepwise elimination procedure based on the Wald test, with a significance threshold of α=0.05 for variable removal. This iterative process resulted in the retention of four genes—*IGHM*, *NEAT1*, *IL32*, and *ACTG1*—as the final predictors in the model. The multinomial logistic regression model was constructed according to the following formula:

Group ∼ *IGHM* + *NEAT1* + *IL32* + *ACTG1*.

where Group denotes the three-category outcome variable (Normal/GMPP/RMPP), and the four genes were treated as continuous predictors.​ The model estimates the probability of each sample belonging to each of the three categories relative to a specified reference category.

Model calibration was evaluated by comparing predicted probabilities with observed outcomes. Using the fitted four-gene model, class-specific predicted probabilities were obtained for both the training cohort and the temporally split validation cohort. Calibration analysis focused on RMPP as the target outcome. Predicted probabilities of RMPP were stratified into deciles, and within each decile, the mean predicted probability was plotted against the observed proportion of RMPP cases. Calibration curves were visualized along with the 45° identity line, which represents perfect agreement between predicted and observed risks. Additionally, calibration was quantitatively assessed using the Brier score for RMPP prediction within a “one vs rest” framework, defined as the mean squared difference between the predicted probabilities and the observed outcomes.

### 2.8 Dual-dimensional evaluation strategy

To evaluate the model’s discriminative ability across different categories, we employed the macro-average AUC metric. This indicator independently calculates the area under the AUC for each category using the “One-vs-Rest” approach. The final macro-average AUC value represents the arithmetic mean of AUCs across all categories, equally reflecting the model’s comprehensive identification performance for all classes.

To assess clinical utility, overall diagnostic accuracy was calculated. Optimal decision thresholds for each category were first determined based on the Youden index, which maximizes the sum of sensitivity and specificity. Threshold determination and ROC analysis were performed using the pROC package (version 1.18.5). When samples exceeded thresholds for multiple categories, class assignment followed clinical priority rules (Normal > GMPP > RMPP). The final accuracy was defined as the proportion of correctly classified samples, reflecting the model’s performance in practical diagnostic scenarios.

### 2.9 External and internal validation of the multi-gene model

For external validation, an independent, prospectively recruited cohort (18 Normal, 19 GMPP, and 17 RMPP subjects) served as the validation set. ROC curves were plotted, and the AUC for each class were calculated to assess the model’s generalizability to unseen temporal data.

For internal validation, all available samples (the combined training and validation cohorts) were utilized to construct a full-dataset model following the same variable selection and model-building procedures. The stability of this model was then evaluated via 1000 iterations of stratified bootstrap resampling. In each iteration, a sample of size n=349 was drawn with replacement from the full dataset, and the multinomial logistic regression model was refitted using the same four-gene signature. The sensitivity at 100 predefined false positive rate points (ranging from 0.00 to 1.00 in increments of 0.01) was recorded for each ROC curve. The distribution of sensitivity values at each false positive rate point across all bootstrap iterations was visualized using boxplots, with the average ROC curve superimposed to illustrate the central tendency of model performance.

### 2.10 Statistical software and packages

All statistical analyses were performed using R software (version 4.3.0). The following packages were employed for specific analyses: nnet for multinomial logistic regression modeling, pROC for ROC curve analysis and AUC calculations, tidyverse (v2.0.0) for data manipulation and visualization, rstatix for statistical testing, and patchwork for combining multiple plots. The bootstrap validation procedure was implemented using base R functions along with dplyr for data processing.

## 3 Results

### 3.1 Study population and single-cell transcriptional profiling

**Figure 1:**
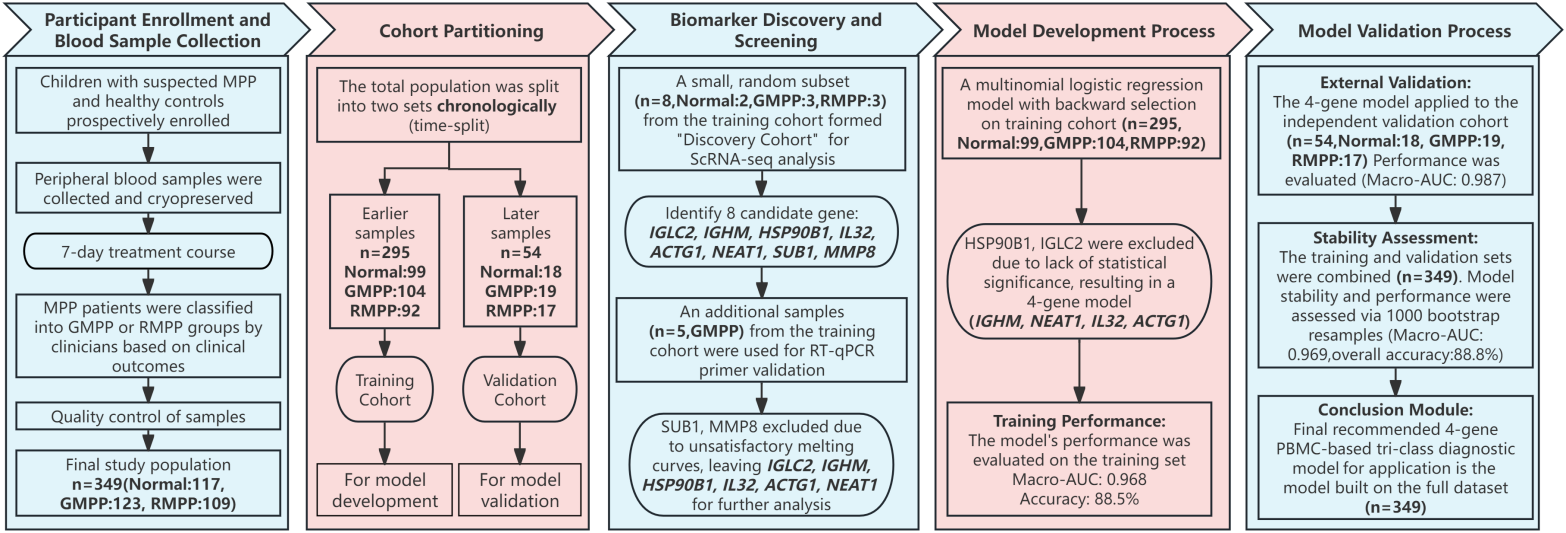
Study flow chart.

Our study included a total of 349 children (117 Normal, 123 GMPP, and 109 RMPP), who were chronologically divided into a training cohort (n=295) for model development and a validation cohort (n=54) for external validation (**Figure 1**). Age distributions across the three groups were non-normal with unequal variances; therefore, intergroup comparisons were conducted using the Kruskal-Wallis test. The results indicated no significant difference in median age (GMPP: 5.7 years, Normal: 5.9 years, RMPP: 5.83 years, p= 0.942). Similarly, sex distribution was compared using the Chi-squared test, which revealed no statistically significant difference (GMPP: 51.2% male, Normal: 54.7% male, RMPP: 50.5% male; p = 0.790).

**Figure 2:**
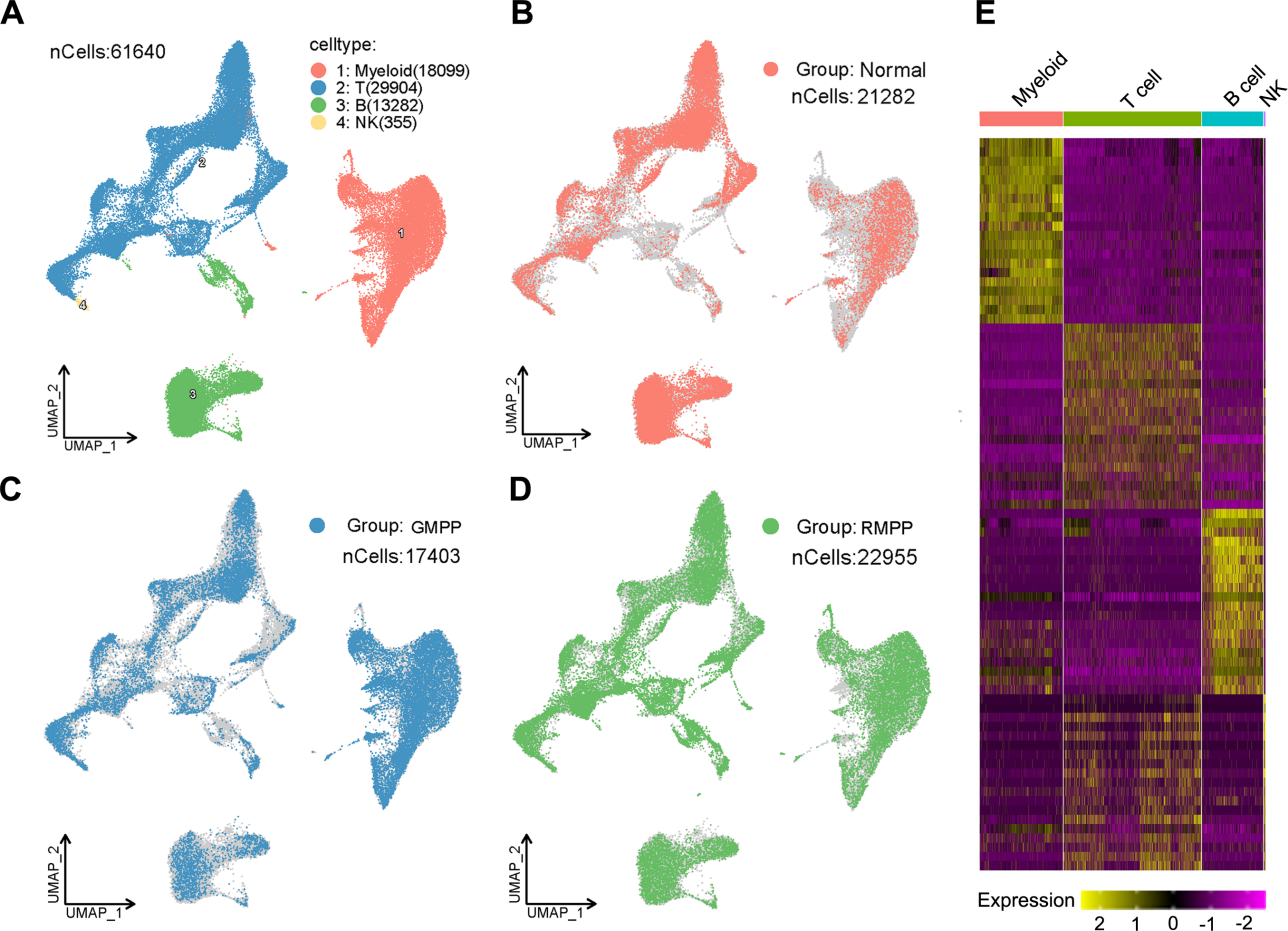
scRNA-seq atlas of PBMCs from normal, GMPP, and RMPP groups. **(A)** UMAP visualization of the integrated single-cell transcriptomic profiles from eight samples after clustering and cell type annotation. **(B-D)** UMAP plots displaying the cellular distribution for each group separately. **(E)** Heatmap illustrating the highly expressed genes specific to each of the four major cell types.

To delineate the specific transcriptional profile of PBMCs and identify candidate biomarkers, we began by performing scRNA-seq on a discovery cohort, which was formed by randomly selecting eight subjects from the training cohort (3 Normal, 3 GMPP, and 3 RMPP). One Normal group sample was excluded due to inadequate cell quality, while the remaining eight samples met the quality criteria (**Supplementary Figure 1**), yielding a total of 61,640 high-quality cells (**Figure 2A**). The cellular composition across groups was as follows: 21,282 cells (34.53%) from the Normal group, 17,403 cells (28.23%) from the GMPP group, and 22,955 cells (37.24%) from the RMPP group (**Figures 2B–D**). Unsupervised clustering categorized all cells into four distinct clusters. Each cluster was assigned a cell identity based on the expression of canonical cell-type markers (**Supplementary Figure 2**), resulting in the identification of 18,099 myeloid cells (expressing VCAN and AIF1), 29,904 T cells (expressing CD3E and CD3D), 13,282 B cells (expressing CD79A and MS4A1), and 355 NK cells (expressing XCL2 and NCAM1) (**Figure 2A**). The cellular distribution for each group (Normal, GMPP, and RMPP) was individually visualized using UMAP projection (**Figures 2B–D**). To further investigate the functional characteristics of each cell population, we performed functional enrichment analysis on the specifically highly expressed genes in each cell type (**Supplementary Table 1**; **Figure 2E**; **Supplementary Figure 3**). The results indicated that myeloid cell-specific genes were significantly enriched in processes such as myeloid leukocyte activation and response to interferon-gamma. T cell-specific genes were associated with the T cell receptor signaling pathway and antigen receptor-mediated signaling pathway. B cell-specific genes were primarily involved in B cell-mediated immunity and immunoglobulin-mediated immune response. Meanwhile, NK cell-specific genes were enriched in natural killer cell activation and natural killer cell-mediated cytotoxicity.

### 3.2 Screening of potential marker genes for RMPP

**Figure 3:**
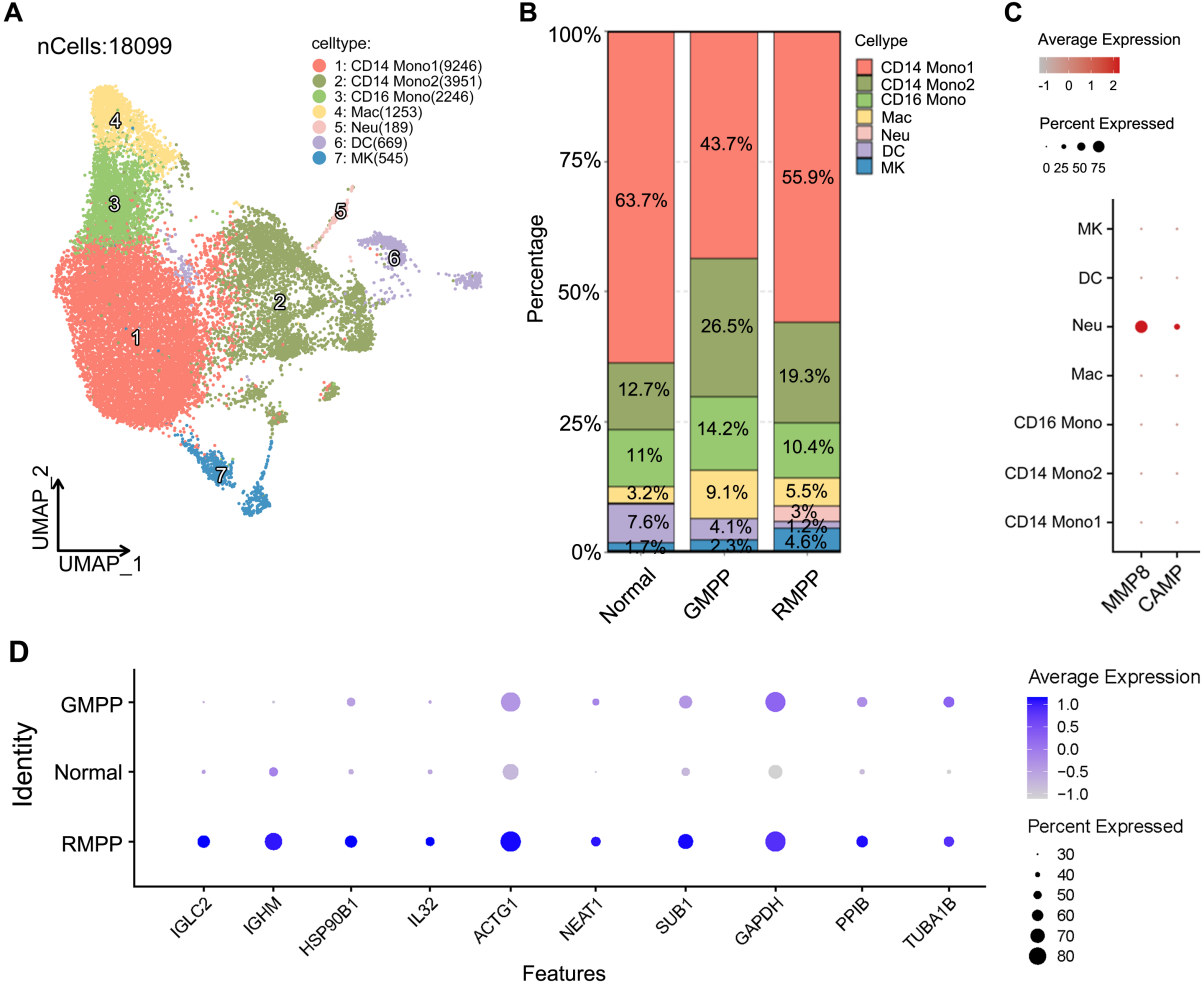
Screening of potential marker genes for RMPP. **(A)** UMAP plot illustrating the seven subclusters of Myeloid cells. **(B)** Proportions of cell subclusters. **(C)** Marker genes for myeloid cell subsets. **(D)** Genes specifically overexpressed in the RMPP groups.

To identify potential marker genes for RMPP, we conducted a detailed analysis of myeloid cells, T cells, B cells, and NK cells. Each cell type was further subdivided into distinct subsets, and alterations in the proportions of these subsets were compared among the Normal, GMPP, and RMPP groups. We observed that among the seven identified subtypes of myeloid cells (CD14 Mono1, CD14 Mono2, CD14 Mono3, Mac, Neu, DC, and MK) (**Figure 3A**), only the Neu subtype exhibited an increased proportion in the RMPP group (**Figure 3B**). No evident changes in cellular proportions were observed among the subsets of the other three cell types. Consequently, *MMP8*, a marker gene of the Neu subpopulation (**Figure 3C**, **Supplementary Figure 4**), was selected as a potential RMPP marker for subsequent validation.

Furthermore, comparative gene expression analysis identified a set of genes specifically highly expressed in the RMPP group compared to the GMPP and Normal groups, including *IGLC2*, *IGHM*, *HSP90B1*, *IL32*, *ACTG1*, *NEAT1*, *SUB1*, *PPIB*, and *TUBA1B* (**Figure 3C**, **Supplementary Table 1**). In summary, through systematic single-cell data analysis, we identified *MMP8*, *IGLC2*, *IGHM*, *HSP90B1*, *IL32*, *ACTG1*, *NEAT1*, and *SUB1* as potential marker genes capable of distinguishing GMPP from RMPP.

### 3.3 Validation of DEGs

To validate the candidate genes, we first performed a technical validation of the RT-qPCR assays. RNA from 5 randomly selected GMPP samples was used to measure the mRNA expression levels of the eight candidate genes (*IGLC2*, *IGHM*, *HSP90B1*, *IL32*, *ACTG1*, *NEAT1*, *SUB1*, and *MMP8*) using RT-qPCR. Preliminary results indicated that the primers for *SUB1* and *MMP8* produced non-specific amplification (**Supplementary Figure 5**), leading to their exclusion from further analysis.

**Figure 4:**
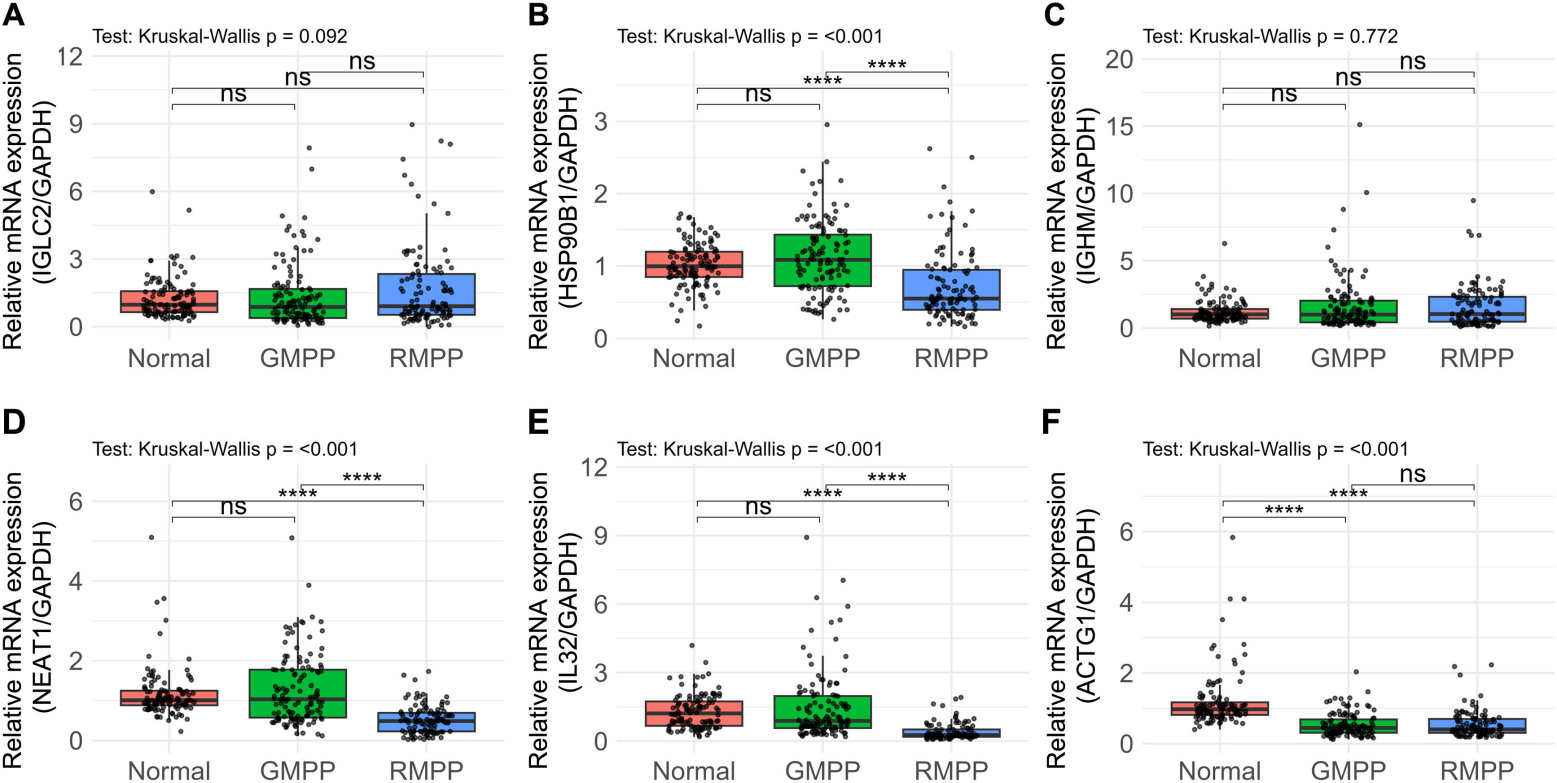
The mRNA expression levels of the six candidate marker genes detected by RT-qPCR in the full-dataset. **(A)** *IGLC2*. **(B)** *HSP90B1*. **(C)** *IGHM*. **(D)** *NEAT1*. **(E)** *IL32*. **(F)** *ACTG1*. ****p<0.001.

We then quantified the mRNA levels of the remaining six genes (*IGLC2*, *IGHM*, *HSP90B1*, *IL32*, *ACTG1*, *NEAT1*) in the entire study cohort (N = 349). As the expression data deviated from normality (Shapiro-Wilk test, P<0.05), the Kruskal-Wallis test was used to assess inter-group differences. The expression levels of four genes—*HSP90B1*, *IL32*, *ACTG1*, and *NEAT1*—were significantly different across the Normal, GMPP, and RMPP groups (**Figure 4**).

### 3.4 Construction and training performance of the three-category diagnostic model

**Figure 5:**
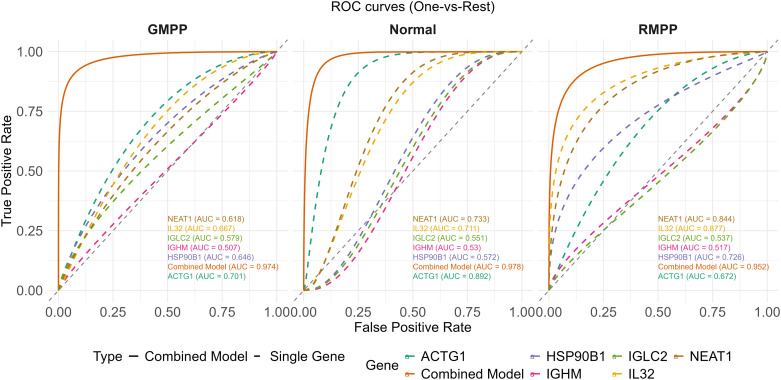
ROC curves of single-gene models and the four-gene combined model in the three-class classification.

We began by evaluating the individual discriminative capacity of the six genes for the three-category outcome (Normal/GMPP/RMPP) using univariable multinomial logistic regression models with a “One-vs-Rest” strategy in the training cohort (n=295). Smoothed ROC curves were plotted for each comparison. The AUC values for each gene varied across the categories: (1) Normal vs Rest: *ACTG1* (0.892) > *NEAT1* (0.733) > *IL32* (0.711) > *HSP90B1* (0.572) > *IGLC2* (0.551) > *IGHM* (0.530); (2) GMPP vs Rest: *ACTG1* (0.701) > *IL32* (0.667) > *HSP90B1* (0.646) > *NEAT1* (0.618) > *IGLC2* (0.579) > *IGHM* (0.507); (3) RMPP vs Rest: *IL32* (0.877) > *NEAT1* (0.844) > *HSP90B1* (0.726) > *ACTG1* (0.672) > *IGLC2* (0.537) > *IGHM* (0.517). These results indicate varying discriminative abilities of these genes among the three categories (**Figure 5**, **Table 2**).

**Table 2:** Coefficients of four-gene combined three-class multivariate logistic model from the training dataset.

Dependent variable category	Predictor variable	β coefficient (95% CI)	P value
GMPP	Intercept	4.800 (3.095 – 6.504)	<0.0001
	*IGHM*	2.424 (1.439 –3.408)	<0.0001
	*NEAT1*	0.994 (0.081 – 1.907)	0.0329
	*IL32*	3.286 (1.960 – 4.611	<0.0001
	*ACTG1*	-17.750 (-22.224 – -13.276)	<0.0001
RMPP	Intercept	5.706 (3.951 – 7.462)	<0.0001
	*IGHM*	3.103 (2.084 – 4.123)	<0.0001
	*NEAT1*	-2.419 (-3.718 – -1.120)	0.003
	*IL32*	-5.327 (-7.198 – -3.457)	<0.0001
	*ACTG1*	-4.759 (-7.153 – -2.366)	<0.0001

**Figure 6:**
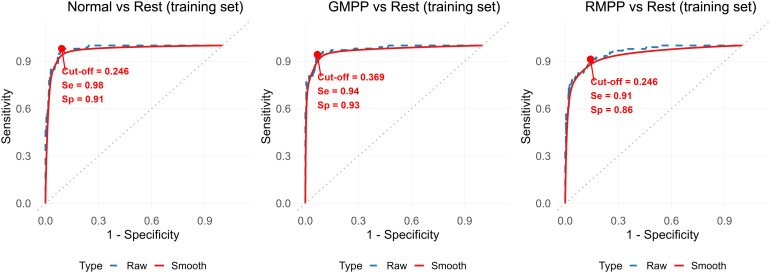
Optimal cut-off values, sensitivity, and specificity from the original ROC curves of the combined model from the training set.

To improve diagnostic performance, we developed a multi-gene combined model. A multi-variable multinomial model with backward stepwise selection retained four genes—*IGHM*, *NEAT1*, *IL32*, and *ACTG1*—as independent diagnostic factors (all P < 0.05). This four-gene combined model demonstrated superior performance, with “One-vs-Rest” AUCs of 0.978 (Normal vs. Rest), 0.974 (GMPP vs. Rest), and 0.952 (RMPP vs. Rest), and a macro-average AUC of 0.968, significantly outperforming any single gene (**Figure 5**, **Table 3**). Using the maximum Youden index, optimal cut-off values were determined (**Figure 6**), yielding an overall diagnostic accuracy of 88.5% in the training set.

**Table 3:** Coefficients of four-gene combined three-class multivariate logistic model from the full dataset.

Dependent variable category	Predictor variable	β coefficient (95%CI)	P value
GMPP	Intercept	4.747 (3.120 – 6.373)	<0.0001
	*IGHM*	2.593 (1.632 – 3.553)	<0.0001
	*NEAT1*	1.191 (0.354 – 2.028)	0.005
	*IL32*	3.588 (2.261 – 4.915)	<0.0001
	*ACTG1*	-19.003 (-23.419 – -14.587)	<0.0001
RMPP	Intercept	5.869 (4.191 – 7.546)	<0.0001
	*IGHM*	3.225 (2.231 – 4.220)	<0.0001
	*NEAT1*	-2.429 (-3.667 – -1.190)	<0.0001
	*IL32*	-5.245 (-7.006 – -3.483)	<0.0001
	*ACTG1*	-5.346 (-7.711 – -2.980)	<0.0001

### 3.5 External validation and full-dataset model construction

**Figure 7:**
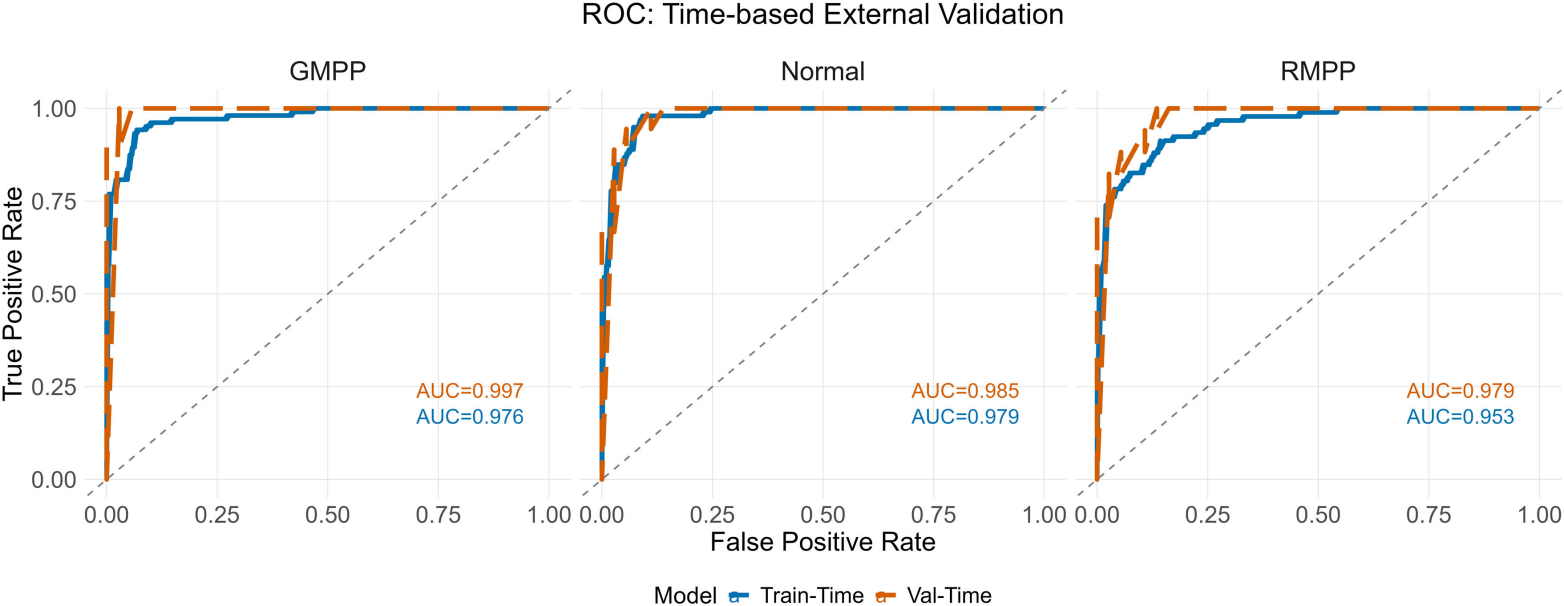
Comparison of ROC curves between the training cohort and the validation cohort.

The model’s generalizability was assessed in an independent, temporally split validation cohort (n=54). It maintained excellent discriminatory power with AUCs of 0.985 (Normal vs. Rest), 0.997 (GMPP vs. Rest), and 0.979 (RMPP vs. Rest) (**Figure 7**). The macro-average AUC reached 0.987, indicating robust performance without significant decay and confirming the model’s strong generalizability to unseen temporal data.

**Figure 8:**
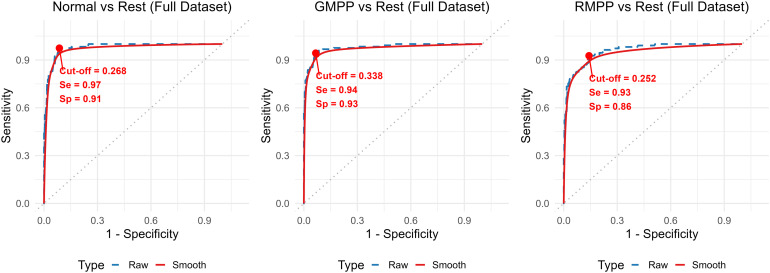
Optimal cut-off values, sensitivity, and specificity from the original ROC curves of the combined model from the full-dataset.

To enhance model stability by leveraging the maximum available data, we constructed a full-dataset model using the entire dataset (n=349), following the identical variable selection procedure. The same four genes were retained, and the model demonstrated excellent diagnostic performance in discriminating among Normal, GMPP, and RMPP, achieving an overall accuracy of 88.8%. The optimal cut-off values were determined using the Youden index and the sensitivity for each category was 97%, 94%, and 91%, respectively, while the specificity was 91%, 93%, and 86%, respectively. (**Figure 8**).

### 3.6 Calibration of the four-gene multinomial diagnostic model

The calibration performance of the four-gene multinomial logistic regression model was evaluated by comparing predicted probabilities with observed outcomes, with a focus on RMPP as the target category. Predicted probabilities were grouped into deciles for analysis.

**Figure 9:**
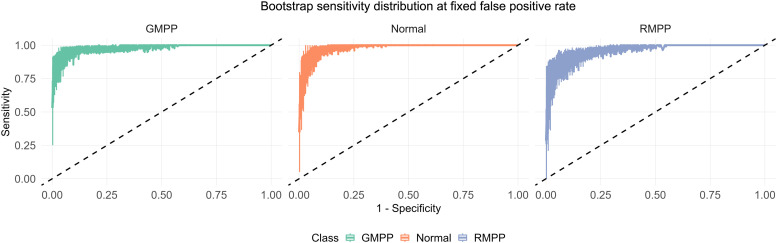
Calibration of the four-gene combined model.

In the training cohort, the calibration curve closely approximated the 45° line of perfect calibration across the entire probability range, indicating excellent agreement between predicted and observed RMPP risks. Only minor deviations were noted at the probability extremes, suggesting negligible overestimation or underestimation of risk (**Figure 9**).

In the temporally split validation cohort, the calibration curve exhibited a generally monotonic pattern, confirming that higher predicted probabilities corresponded consistently to higher observed frequencies of RMPP. At low predicted probability levels, the observed RMPP rate remained near zero, reflecting a low false-positive risk among patients classified as low probability. At higher probability levels, the observed event rate slightly exceeded the predicted risk, indicating a modest underestimation of RMPP risk in the highest-risk subgroup. Overall, these calibration results support the reliability and generalizability of the four-gene model for estimating individual-level RMPP probabilities.

In addition to graphical calibration assessment, the Brier score for RMPP prediction under a “one vs rest” framework was 0.077 in the training cohort and 0.055 in the validation cohort, demonstrating good overall accuracy and stable probabilistic calibration.

### 3.7 Internal validation and final model recommendation

**Figure 10:**
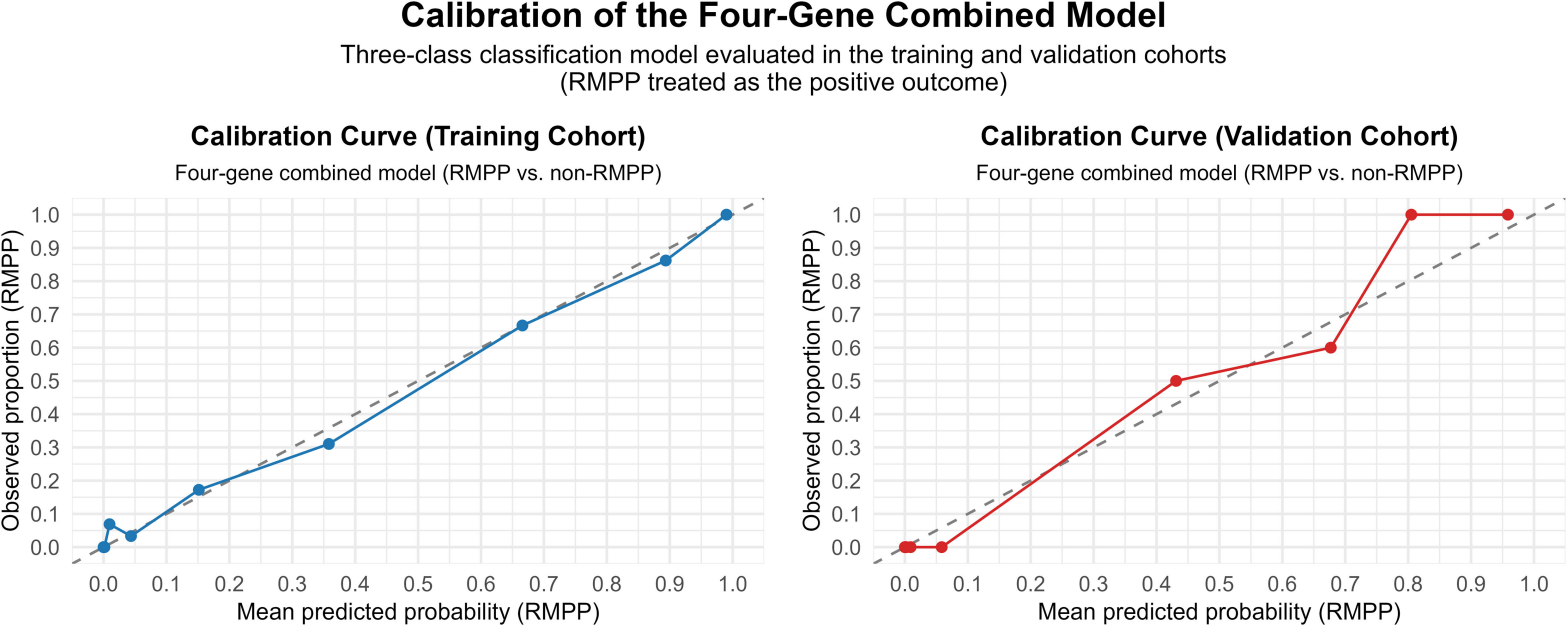
Model stability assessment using 1000 bootstrap resampling.

The stability of the full-dataset model was rigorously evaluated through 1000 iterations of stratified bootstrap resampling (**Table 4**). This internal validation yielded a macro-average AUC of 0.969 (95%CI:0.947-0.988). A “Boxplot-Average Curve” plot visualized the robust and stable performance across bootstrap samples (**Figure 10**).

**Table 4:** Internal validation results of the four-gene combined model based on 1000 bootstrap resamples.

Category	AUC (95%CI)	Cut-off (95%CI)	Sensitivity (95%CI)	Specificity (95%CI)
Normal	0.977 (0.960–0.993)	0.307 (0.163–0.535)	0.963 (0.940–0.985)	0.921 (0.890–0.950)
GMPP	0.974 (0.958–0.992)	0.375 (0.201–0.592)	0.931 (0.905–0.960)	0.937 (0.910–0.960)
RMPP	0.956 (0.914–0.985)	0.388 (0.161–0.622)	0.904 (0.870–0.940)	0.906 (0.880–0.930)

A comparison of the regression coefficients between the training-set model and the full-dataset model revealed only minor numerical differences, while the signs (directions of effect) remained entirely consistent (**Table 5**). This indicates remarkable model stability. Given that the full-dataset model is derived from the entire dataset (n=349), it provides more precise and stable coefficient estimates. Therefore, we recommend the full-dataset model as the final version for potential clinical application. Its overall diagnostic accuracy was 88.8%, demonstrating enhanced statistical power and reliability. In conclusion, the consistent model structure, independent external validation, and rigorous internal bootstrap resampling collectively confirm that the four-gene diagnostic model possesses robust performance and strong generalizability.

**Table 5:** Comparison of model coefficients between the training model and the full-dataset model.

Category	Variable	Training set model coefficient	Full-dataset model coefficient
GMPP	*IGHM*	2.42	2.59
	*NEAT1*	0.994	1.10
	*IL32*	3.29	3.59
	*ACTG1*	-17.7	-19.0
RMPP	*IGHM*	3.10	3.23
	*NEAT1*	-2.42	-2.43
	*IL32*	-5.33	-5.24
	*ACTG1*	-4.76	-5.35

## 4 Discussion

MPP is a common CAP in children, predominantly affecting school-aged children. While often self-limiting, a subset of patients can progress to RMPP. The early diagnosis of RMPP is challenging, its progression can be rapid, and it is frequently associated with extrapulmonary complications, posing significant difficulties in clinical management. The definitive diagnosis of MPP primarily relies on the detection of serum-specific antibodies and pathogen testing (e.g., nucleic acid detection) from nasopharyngeal swabs. However, serum antibodies appear relatively late in the infection course, and obtaining adequate nasopharyngeal swab samples can be difficult in young children due to poor cooperation. Consequently, the diagnosis of RMPP is often based on lagging indicators, such as the progression of clinical symptoms and radiological findings after MPP is confirmed, highlighting the lack of reliable early biological markers. Substantial evidence identifies macrolide resistance as a key factor in RMPP development (Tong et al., 2022; Waites et al., 2017). Both whole-genome sequencing (Chen et al., 2024; Jiao et al., 2025) and metagenomic next-generation sequencing (Xu et al., 2024; Lin et al., 2023) have confirmed that the A2063G point mutation in the 23S rRNA gene is the predominant resistance mechanism, although other mutations beyond A2063G and A2064G exist. A multiplex amplicon sequencing technique developed by Japanese researchers enables simultaneous MLST typing, P1 typing, and resistance locus analysis (Kubota et al., 2025), but current assay kits offer limited coverage of other potential mutation sites. Notably, some studies report no significant correlation between macrolide resistance gene mutations and the occurrence of RMPP (Jiang et al., 2025; Xu et al., 2021; Zhan et al., 2022; He et al., 2024), indicating that the relationship between resistance and RMPP requires further elucidation.

This study presents, for the first time, the peripheral blood immune cell transcriptomic landscape at single-cell resolution from children in the Normal, GMPP, and RMPP groups within 24 hours of hospital admission, ensuring that the identified biomarkers reflect the early disease state. By analyzing differences in immune cell subset proportions and functional states among these groups, we identified eight genes specifically highly expressed in RMPP: *IGLC2*, *IGHM*, *HSP90B1*, *IL32*, *ACTG1*, *NEAT1*, *SUB1*, and *MMP8*. The expression trends of these genes were subsequently confirmed via RT-qPCR in a prospective independent cohort. Furthermore, we constructed a combined diagnostic model utilizing *IGHM*, *NEAT1*, *IL32*, and *ACTG1*. This model effectively discriminates among the three categories (Normal/GMPP/RMPP) with clear and reliable results. However, it is noteworthy that the detection of biomarker expression levels in PBMCs via RT-qPCR relies on a relatively complex and rigorously standardized sample processing procedure, which may limit its potential for immediate clinical translation. Nonetheless, this model offering a novel molecular diagnostic tool to address the challenge of early RMPP identification and holding significant potential for clinical application. RMPP represents the most severe clinical phenotype, characterized by rapid progression and an increased risk of complications, making it the primary target for early risk stratification. Although our model performs three-class classification, particular emphasis was placed on its ability to accurately identify RMPP, which is most clinically relevant for decision-making. The consistently low Brier scores in both the training and validation cohorts further support the reliability of the model’s probabilistic predictions and indicate no substantial overfitting.

Various clinical prediction models for RMPP have been reported (Jiang et al., 2025; Li et al., 2023; Mu et al., 2025; Wu et al., 2025). Numerous studies have indicated significant associations between inflammatory markers such as LDH, D-dimer, IL6, and the neutrophil-to-lymphocyte ratio with RMPP (Zhang et al., 2016; Fan et al., 2023; Li et al., 2023; Xi et al., 2024). Our previous retrospective study involving 244 MPP children established a prediction model combining bronchoscopy and CT scores, achieving an AUC of 0.82 for discriminating RMPP (Lu et al., 2024). Additionally, we developed a nomogram model based on six independent risk factors—age, APACHE II score, total CT score, secretion color, mucosal edema, and PCT—which yielded AUCs of 0.913 and 0.811 in the development and validation cohorts, respectively (Wu et al., 2025). Another study constructed a prediction model for MPP severity based on four proteins, including CD209 (Liang et al., 2025). Proteomic studies have also suggested proteins like IL33 and HSP90AA1 as potential biomarkers for MPP (Li et al., 2014; Wei et al., 2021). However, these indicators often suffer from limitations such as undefined thresholds, retrospective design, limited variables, single-center validation, or small sample sizes. Transcriptomic approaches offer a promising alternative for assessing pneumonia severity and etiology. For instance, the Molecular Distance to Health (MDTH) score, derived from host transcriptional profiles, enables discrimination of disease severity in pediatric CAP caused by diverse pathogens including MP (Wallihan et al., 2018). Furthermore, pathogen-specific host transcriptomic signatures identified through methods like LASSO regression allow for accurate differentiation of MPP from other bacterial and viral pneumonias, as demonstrated by validation in independent cohorts (Viz-Lasheras et al., 2025a). The multi-gene diagnostic model developed in this study integrates genes involved in multiple immune pathways. It shows promise for identifying children at high risk for RMPP early in the disease course, potentially even before characteristic radiological changes manifest, by detecting alterations in peripheral blood gene expression. This could facilitate early intervention and rational allocation of healthcare resources. For children identified as high-risk by this model, more aggressive immunomodulatory therapies, such as corticosteroids, could be considered to prevent progression to RMPP. Currently, RT-qPCR based on throat swabs is the mainstream rapid technique for MP detection. However, poor tolerance to swab sampling in young children, the primary demographic for MPP, often leads to suboptimal sample quality, increasing the risk of false-negative results, potential treatment delays, and possibly contributing to RMPP occurrence. The three-category diagnostic model developed in this study could partially circumvent the issue of false negatives from poor swab sampling. Furthermore, it enables screening and typing within a single assay, offering greater clinical utility compared to traditional binary classification models. For optimal diagnostic accuracy, the output of this three-category model should be integrated with clinical diagnostic findings, thereby optimizing the distinction between GMPP and RMPP.

Omics technologies have deepened the understanding of MPP from multiple dimensions, with several studies integrating various omics approaches to reveal the pathogenic mechanisms of MP (Liang et al., 2025; Li et al., 2014; Tang et al., 2025; Liao et al., 2025; Iannuzo et al., 2023). Through single-cell RNA sequencing, we observed at the transcriptional level that aberrant activation and chemotaxis of innate immune cells, such as Neu subtype, may be a key mechanism in RMPP. The upregulation of specific genes likely promotes the excessive recruitment and infiltration of these inflammatory cells into the lungs, exacerbating pulmonary inflammation severity. This aligns with the prevailing view that RMPP pathogenesis is driven more by an excessive immune-inflammatory response and immune dysregulation following infection than by direct damage from MP itself (Tong et al., 2022; Wang et al., 2014). In this study, single cell sequencing revealed a marked increase in the Neu subtype in peripheral blood of RMPP patients, and *MMP8* is a marker gene of this subtype, suggesting that *MMP8* could be a potential biomarker for RMPP. Unfortunately, due to primer specificity issues, we were unable to validate *MMP8* in a larger sample set. Several studies have examined peripheral blood *MMP8* levels in pneumonia patients. Compared with no-CAP patients or healthy controls, *MMP8* expression was elevated in blood cells from CAP patients (Scicluna et al., 2015; Viz-Lasheras et al., 2025a). In pediatric pneumonia, whole blood *MMP8* levels were significantly higher in bacterial than in viral pneumonia (Viz-Lasheras et al., 2025a). Moreover, while plasma *MMP8* levels and whole blood *MMP8* gene expression were significantly higher in CAP patients than in non-infectious respiratory failure patients and healthy volunteers, its diagnostic specificity when used alone remains limited (Uhel et al., 2019). Future studies should explore its additive value as part of a multi marker panel.

The four genes identified in our diagnostic model may cooperatively contribute to the pathogenesis of RMPP through distinct mechanisms. First, the specific upregulation of *NEAT1* in peripheral blood cells from children with RMPP suggests its role as a key regulatory hub, potentially activating the JNK/NLRP3 or PTBP1/FOXP1 signaling axes to promote M1 macrophage polarization and the release of pro-inflammatory cytokines, thereby exacerbating pulmonary inflammation (Huang et al., 2025; Liu et al., 2024). Concurrently, the persistent high expression of *IL32* during the convalescent phase of severe infection implies that this cytokine plays a central role in the perpetuation of inflammation in RMPP. *IL32* may drive an excessive inflammatory cascade by inducing a positive feedback loop involving TNF-α and IL-1β (Tang et al., 2020; Zhang et al., 2022). Within this inflammatory network, *NEAT1*-driven NLRP3 inflammasome activation may intersect with the pro-inflammatory effects of *IL32*, collectively amplifying immune injury in the airways and lungs. Moreover, aberrant expression of the humoral immunity-related gene *IGHM* could compromise early host defense mechanisms, impair effective pathogen clearance, and thereby create a permissive environment for persistent inflammation (Li et al., 2024; Ademokun et al., 2011). Meanwhile, *ACTG1* may not only exacerbate immune dysregulation through PI3K/Akt-mediated apoptosis of immune cells but also influence immune cell migration via its involvement in cytoskeletal remodeling during infection, potentially contributing to the dissemination of pulmonary lesions and disease prolongation (Yao et al., 2023; Parry et al., 2025). Collectively, these four molecules constitute a complex regulatory network spanning inflammation initiation and amplification (*NEAT1*, *IL32*), humoral immune defense (*IGHM*), and cytoskeletal dynamics/immune homeostasis (*ACTG1*), which together promote the hyperinflammatory response and disease progression in RMPP. This multifaceted involvement underscores their potential combined value as early diagnostic biomarkers. Encouragingly, RT-qPCR validation in our prospective cohort confirmed expression trends highly consistent with the scRNA-seq results, supporting their reliability as biomarkers. Further studies could validate the present biomarker signature in external publicly available datasets to strengthen the reliability of our findings, as well as assess the diagnostic utility of this panel of markers in peripheral blood samples from children with pneumonia caused by different pathogens.

This study provides a new strategy for the early diagnosis and precise intervention of RMPP, while also offering clues to its pathogenesis. If validated by larger-scale prospective clinical trials, this model holds promise as a novel liquid biopsy tool. Potential application scenarios include: assisting in the early diagnosis and classification of suspected cases; dynamically monitoring gene expression changes in GMPP patients to warn of potential progression to RMPP; and reducing the false-negative rate in MPP diagnosis. However, this study has several limitations. First, the sample size for scRNA-seq was relatively small due to the current high cost of the technology, which may limit the statistical power and precision of biomarker discovery. Second, the specimens for the prospective validation cohort were sourced from a single center, potentially affecting the generalizability of the findings. Nonetheless, the samples collected from Shenzhen Children’s Hospital, the only tertiary pediatric hospital in Southern China, benefit from the city’s large pediatric population, high MPP incidence, high proportion of young children, and frequent challenges in obtaining adequate pharyngeal swabs from infants, thus offering considerable diversity and representativeness to partially offset the single-center limitation. Third, the limited sample size of the prospective cohort may also impact the general applicability of the results. Finally, due to significant differences in routine clinical test results between the Normal group and the patient groups, the model could not incorporate common clinical indicators. MP infections often coexist with other pathogens in the real-world clinical scenarios. For instance, MP-carrying children show a higher rate of viral co-detection than non-carriers (Koenen et al., 2023). Another study reported that approximately 28% of young children with MP infection had other pathogens—primarily viruses—detected concurrently (Kutty et al., 2019). Moreover, some patients are hospitalized for pneumonia based on clinical criteria even when culture or PCR results are negative (Jain et al., 2015). To better address the complexity of co-infections, future studies should perform scRNA-seq to investigate MPP cases with bacterial co-infection and viral co-infection. Integrating such new sequencing datasets with the existing dataset from non-coinfected MPP cases in this study will enable a systematic comparative analysis. Future research should also expand the scRNA-seq cohort size, validate and refine the model using multi-center cohorts, and explore the feasibility of developing these biomarkers into clinical testing kits. Concurrently, functional studies on core genes, such as *IL32*, are warranted to delve deeper into their molecular mechanisms in RMPP, particularly in corticosteroid resistance, with the aim of identifying novel therapeutic targets.

## 5 Conclusion

In conclusion, this study identifies a distinct peripheral immune gene signature in childhood RMPP through scRNA-seq. We established and validated a robust four-gene (*IGHM*, *NEAT1*, *IL32*, *ACTG1*) three-category diagnostic model using RT-qPCR in prospective cohorts. The multivariable logistic regression model demonstrated high performance in discriminating Normal, GMPP, and RMPP, with excellent macro-average AUCs in training, external validation, and internal bootstrap validation, and a good overall accuracy. This model enables early, non-invasive risk stratification within 24 hours of admission, offering a clinically feasible molecular tool for timely intervention, optimized antibiotic use, and improved management of pediatric MPP, potentially reducing severe complications.

## Data Availability

The data that support the findings of this study are available from the corresponding author upon reasonable request.
