# Supplementary material for: From genes to clinical application: a circulating four-gene signature for early diagnosis model of refractory Mycoplasma pneumoniae pneumonia
Source: Front Cell Infect Microbiol. 2026 Apr 7;16:1741058. doi: 10.3389/fcimb.2026.1741058 (PMC13095701; doi:10.3389/fcimb.2026.1741058)
Supplement: Supplementary file 1 [file DataSheet1.pdf]

Supplementary Material

Figure S1

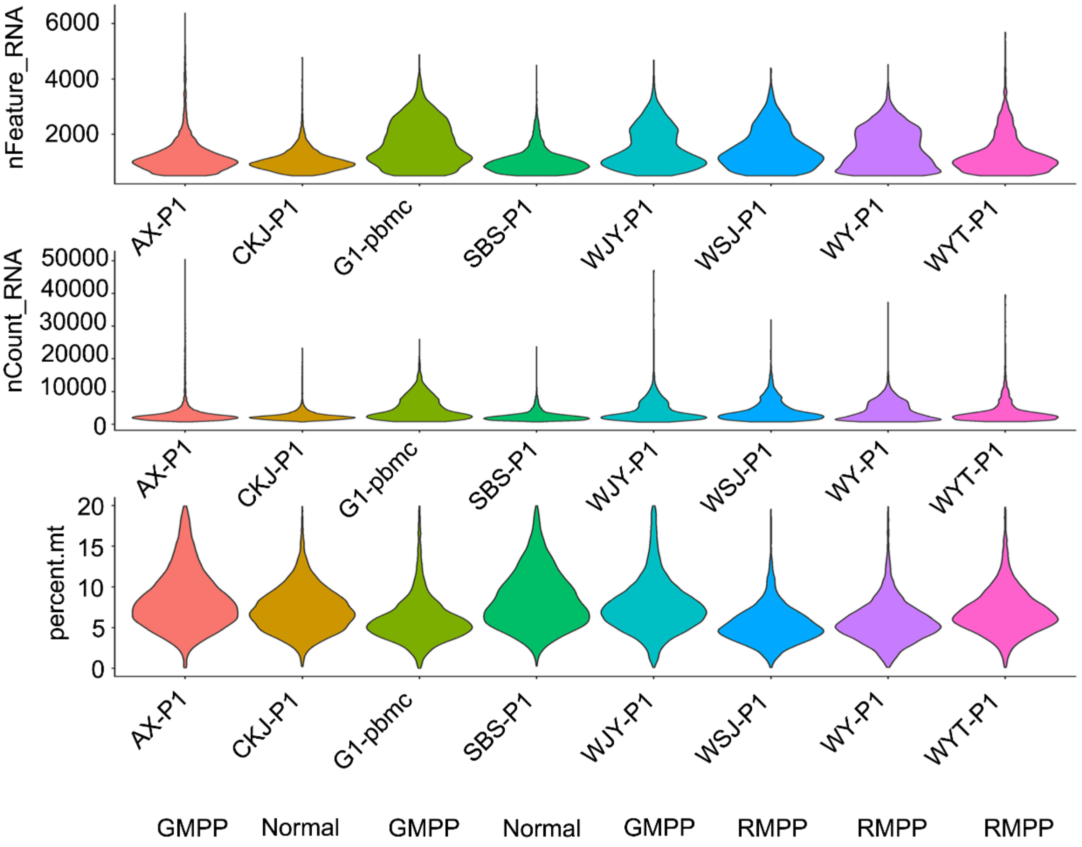

Fig. S1 Data quality control of single-cell RNA sequencing for the eight samples.

Figure S2

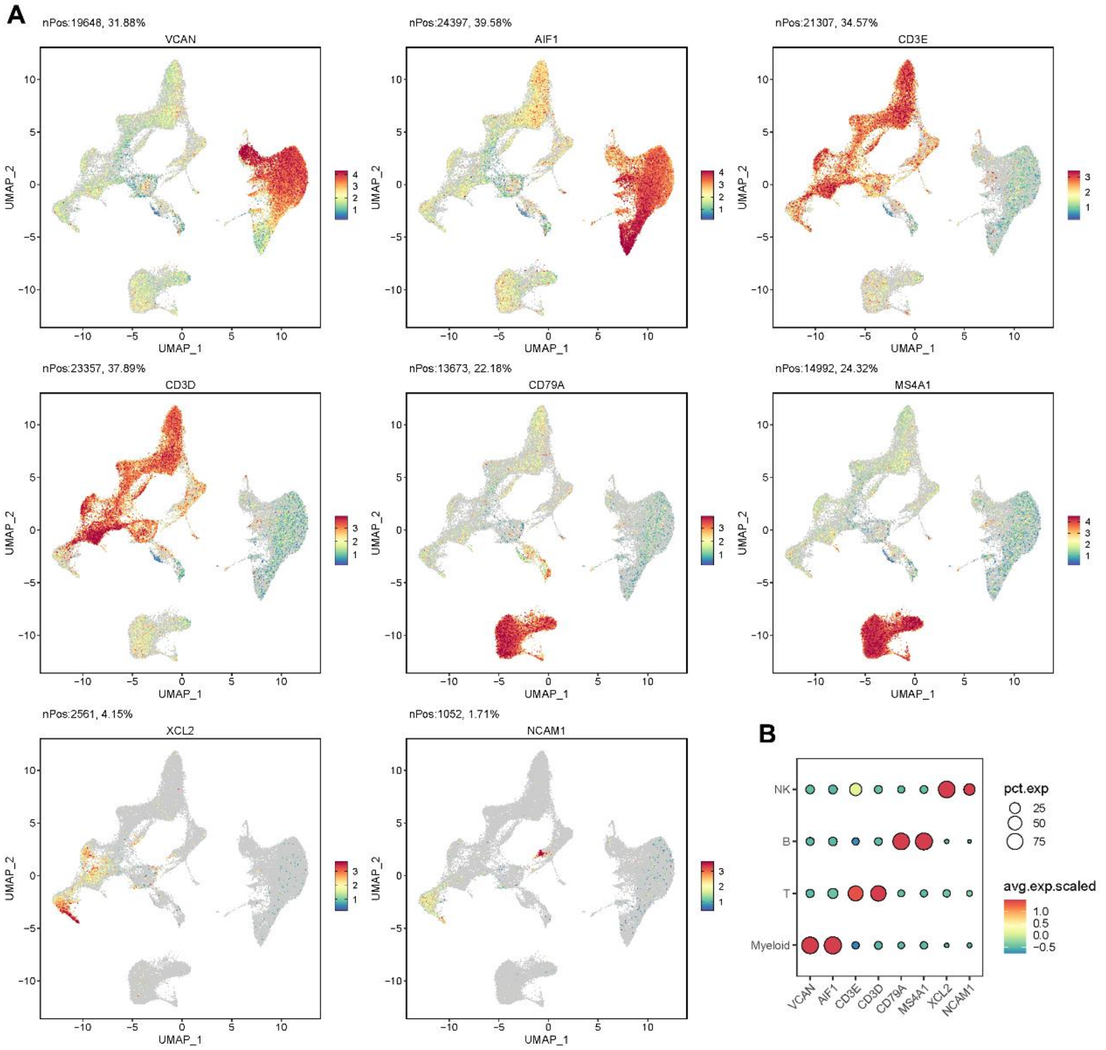

Fig. S2 Marker genes for identified cell types. (A) UMAP visualization of marker gene expression across cell types. (B) Dot plot displaying marker gene expression for each cell type.

Figure S3

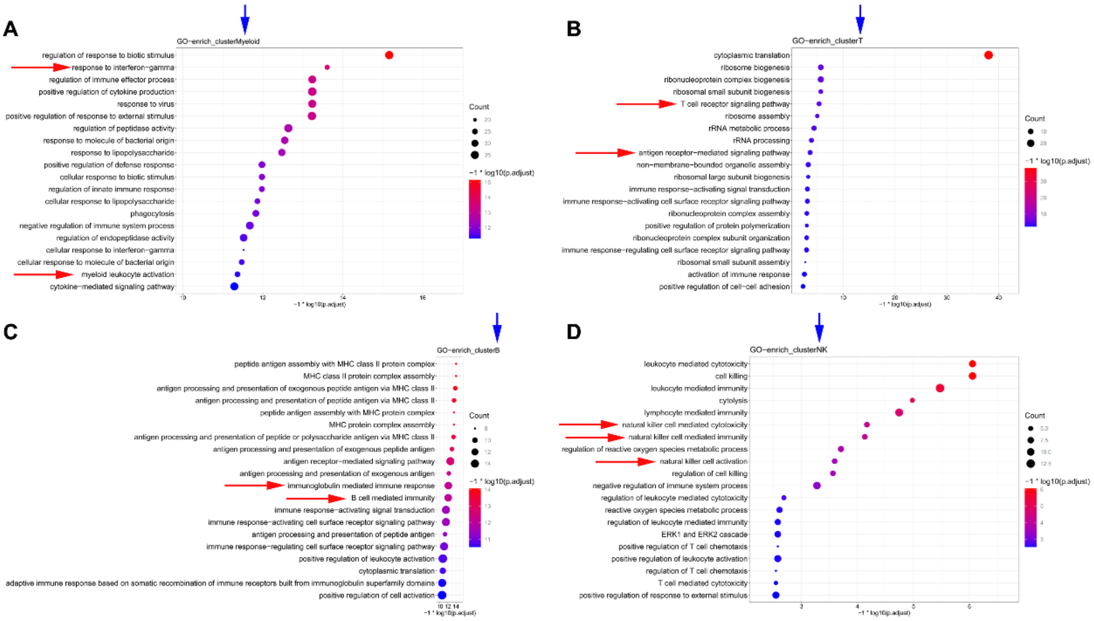

Fig. S3 GO functional enrichment analysis of specifically highly expressed genes in Myeloid cells, T cells, B cells, and NK cells.

Figure S4

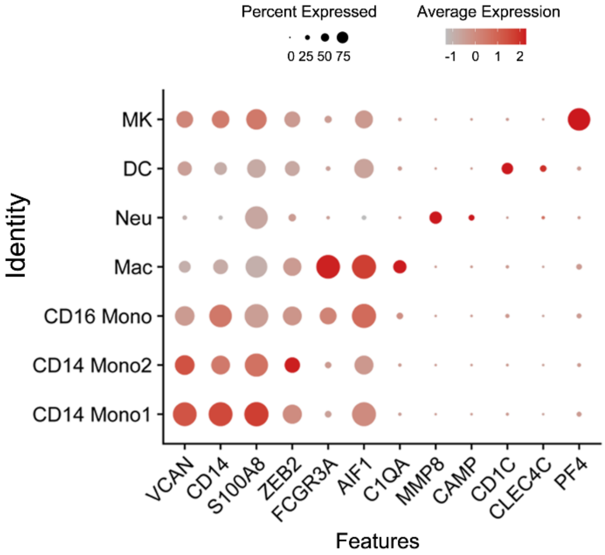

Fig. S4 Marker genes for myeloid cell subsets.

Figure S5

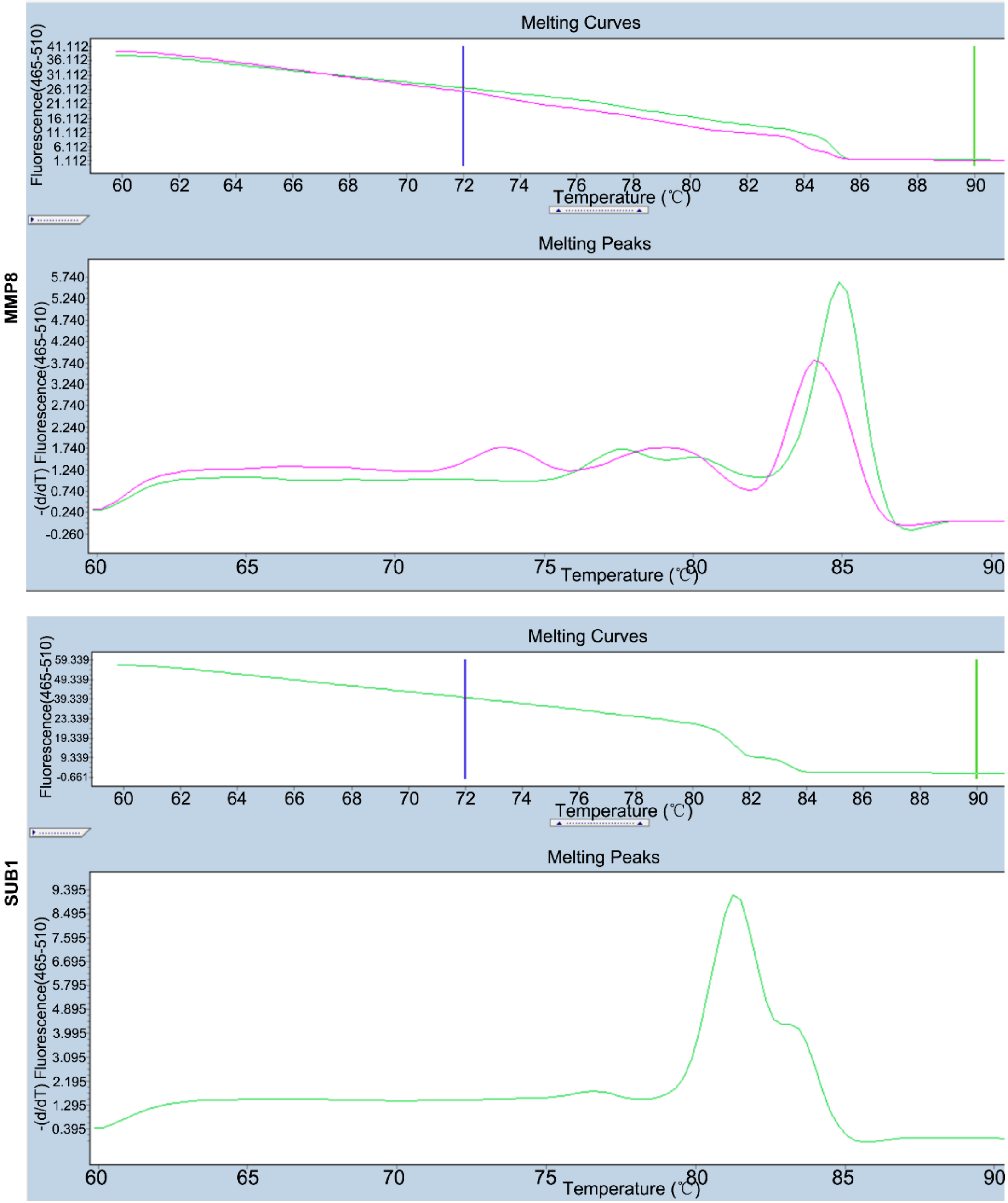

Fig. S5 Amplification and melt curves from RT-qPCR validation of MMP8 and SUB1 gene expression.

Table S1. Lists of differentially expressed genes for each cell type (Myeloid, T, B, NK) (p<0.05).

p_val	avg_log2FC	pct.1	pct.2	p_val_adj	cluster	gene
0	4.861704	0.964	0.412	0	Myeloid	S100A8
0	4.810364	0.972	0.448	0	Myeloid	S100A9
0	4.559659	0.973	0.346	0	Myeloid	LYZ
0	4.050732	0.954	0.162	0	Myeloid	FCN1
0	3.874913	0.963	0.236	0	Myeloid	CST3
0	3.871671	0.931	0.109	0	Myeloid	MNDA
0	3.600358	0.842	0.101	0	Myeloid	VCAN
0	3.590465	0.862	0.103	0	Myeloid	CD14
0	3.540366	0.958	0.341	0	Myeloid	FOS
0	3.382017538	0.707	0.071	0	Myeloid	S100A12
0	3.248587	0.963	0.292	0	Myeloid	TYROBP
0	3.2467	0.913	0.181	0	Myeloid	AIF1
0	3.225113	0.969	0.432	0	Myeloid	CTSS
0	3.214208245	0.87	0.08	0	Myeloid	SERPINA1
0	3.20412	0.906	0.373	0	Myeloid	IFITM3
0	3.080796	0.852	0.069	0	Myeloid	MS4A6A
0	2.957156	0.963	0.397	0	Myeloid	PSAP
0	2.941953	0.867	0.101	0	Myeloid	LST1
0	2.91811	0.952	0.375	0	Myeloid	SAT1
0	2.881852	0.891	0.147	0	Myeloid	GRN
0	2.860425	0.931	0.197	0	Myeloid	FCER1G
0	2.752272	0.832	0.077	0	Myeloid	FGL2
0	2.707008	0.772	0.039	0	Myeloid	FPR1
0	2.696488	0.784	0.047	0	Myeloid	CSTA
0	2.628494	0.768	0.033	0	Myeloid	IGSF6
0	2.607678	0.798	0.036	0	Myeloid	LILRB2
0	2.575512874	0.944	0.393	0	Myeloid	S100A11
0	2.539347	0.799	0.131	0	Myeloid	CEBPD
0	2.513159	0.925	0.344	0	Myeloid	DUSP1
0	2.47417965	0.759	0.041	0	Myeloid	CLEC12A
0	2.465346	0.667	0.032	0	Myeloid	PLBD1
0	2.432974	0.851	0.11	0	Myeloid	SPI1
0	2.425064	0.706	0.087	0	Myeloid	RGS2
0	2.424788	0.991	0.936	0	Myeloid	FTL
0	2.383458	0.746	0.047	0	Myeloid	CPVL
0	2.362049	0.717	0.051	0	Myeloid	DUSP6
0	2.35317	0.713	0.028	0	Myeloid	CSF3R
0	2.352571	0.699	0.037	0	Myeloid	CD36
0	2.339927	0.865	0.213	0	Myeloid	BRI3
0	2.25439	0.76	0.039	0	Myeloid	NCF2
0	2.233263	0.766	0.06	0	Myeloid	HCK
0	2.229497	0.962	0.613	0	Myeloid	S100A6

0	2.225822	0.799	0.215	0	Myeloid	TNFSF10
0	2.194588	0.693	0.031	0	Myeloid	SLC11A1
0	2.15207	0.688	0.03	0	Myeloid	TGFBI
0	2.144677	0.725	0.084	0	Myeloid	LGALS3
0	2.11556	0.819	0.181	0	Myeloid	NPC2
0	2.112416	0.696	0.029	0	Myeloid	TNFAIP2
0	2.099813	0.571	0.016	0	Myeloid	FCGR1A
0	2.098661	0.684	0.035	0	Myeloid	TNFSF13B
0	2.06819	0.738	0.071	0	Myeloid	MPEG1
0	2.05216	0.665	0.032	0	Myeloid	PILRA
0	2.03367	0.783	0.153	0	Myeloid	CTSB
0	2.017246	0.772	0.086	0	Myeloid	RNF130
0	1.99909	0.663	0.024	0	Myeloid	CLEC7A
0	1.991012	0.987	0.917	0	Myeloid	FTH1
0	1.989605	0.849	0.288	0	Myeloid	TSPO
0	1.976664	0.707	0.197	0	Myeloid	IFI6
0	1.976386	0.844	0.252	0	Myeloid	TKT
0	1.976341	0.612	0.082	0	Myeloid	NAMPT
0	1.94809	0.78	0.183	0	Myeloid	CEBPB
0	1.906301	0.967	0.619	0	Myeloid	S100A4
0	1.89368	0.552	0.018	0	Myeloid	MAFB
0	1.851242	0.541	0.023	0	Myeloid	C5AR1
0	1.800432	0.812	0.17	0	Myeloid	FGR
0	1.791345	0.768	0.151	0	Myeloid	APLP2
0	1.785451	0.948	0.458	0	Myeloid	SRGN
0	1.780745	0.874	0.273	0	Myeloid	LGALS1
0	1.775816	0.634	0.121	0	Myeloid	WARS1
0	1.774553	0.677	0.098	0	Myeloid	TIMP1
0	1.771905	0.615	0.019	0	Myeloid	LRP1
0	1.76619	0.578	0.022	0	Myeloid	KLF4
0	1.748232	0.6	0.014	0	Myeloid	LILRB3
0	1.745474	0.579	0.027	0	Myeloid	CFD
0	1.724118	0.694	0.287	0	Myeloid	MT2A
0	1.720572	0.505	0.028	0	Myeloid	PLXDC2
0	1.714886	0.738	0.118	0	Myeloid	JAML
0	1.710166	0.539	0.016	0	Myeloid	CD300E
0	1.703085	0.565	0.022	0	Myeloid	TLR2
0	1.681798	0.566	0.045	0	Myeloid	LRRK2
0	1.667547	0.92	0.521	0	Myeloid	VIM
0	1.661593	0.611	0.089	0	Myeloid	GLUL
0	1.661213	0.874	0.414	0	Myeloid	ZFP36
0	1.660674	0.541	0.016	0	Myeloid	OSCAR
0	1.6534	0.73	0.149	0	Myeloid	ASAH1
0	1.652153	0.573	0.024	0	Myeloid	SLC7A7

0	1.629407	0.557	0.019	0	Myeloid	KCTD12
0	1.625035	0.844	0.297	0	Myeloid	ANXA2
0	1.623304	0.604	0.053	0	Myeloid	CPPED1
0	1.610753	0.695	0.214	0	Myeloid	HLA-DRB5
0	1.609366	0.555	0.016	0	Myeloid	HK3
0	1.608006	0.501	0.032	0	Myeloid	IRAK3
0	1.60003	0.612	0.068	0	Myeloid	GCA
0	1.593836	0.53	0.02	0	Myeloid	PTAFR
0	1.591112	0.508	0.014	0	Myeloid	P2RY13
0	1.589499	0.585	0.048	0	Myeloid	TBXAS1
0	1.58064	0.687	0.177	0	Myeloid	LTA4H
0	1.575149	0.776	0.244	0	Myeloid	TALDO1
0	1.564035	0.709	0.155	0	Myeloid	ZEB2
0	1.562277	0.568	0.039	0	Myeloid	TYMP
0	1.556957	0.654	0.088	0	Myeloid	FCGRT
0	1.556771	0.653	0.098	0	Myeloid	PTPRE
0	1.556285	0.828	0.289	0	Myeloid	VSIR
0	1.550641	0.584	0.051	0	Myeloid	BLVRB
0	1.534967	0.788	0.269	0	Myeloid	C1orf162
0	1.534267	0.543	0.059	0	Myeloid	MEGF9
0	1.530171	0.571	0.045	0	Myeloid	RAB31
0	1.529172	0.758	0.206	0	Myeloid	PYCARD
0	1.520949	0.551	0.029	0	Myeloid	LMO2
0	1.514573	0.571	0.049	0	Myeloid	STX11
0	1.513921	0.799	0.356	0	Myeloid	NFKBIA
0	1.512413	0.533	0.018	0	Myeloid	LRRC25
0	1.508755	0.883	0.396	0	Myeloid	COTL1
0	1.492932	0.599	0.127	0	Myeloid	LAP3
0	1.47879	0.625	0.152	0	Myeloid	DPYD
0	1.478457	0.665	0.137	0	Myeloid	VMP1
0	1.46932	0.641	0.122	0	Myeloid	PLSCR1
0	1.467383	0.743	0.19	0	Myeloid	ANXA5
0	1.457363	0.513	0.039	0	Myeloid	SULT1A1
0	1.455445	0.69	0.122	0	Myeloid	CTSZ
0	1.454998	0.666	0.144	0	Myeloid	AP1S2
0	1.440493	0.529	0.054	0	Myeloid	BLVRA
0	1.41605	0.669	0.293	0	Myeloid	ISG15
0	1.414017	0.856	0.358	0	Myeloid	ANXA1
0	1.408512	0.556	0.066	0	Myeloid	PGD
0	1.396619	0.52	0.03	0	Myeloid	TIMP2
0	1.388919	0.63	0.132	0	Myeloid	STXBP2
0	1.388671	0.756	0.235	0	Myeloid	CARD16
0	1.388202	0.65	0.132	0	Myeloid	LGALS9
0	1.371762	0.753	0.213	0	Myeloid	ZYX

0	1.367601	0.606	0.089	0	Myeloid	NAGK
0	1.366905	0.904	0.491	0	Myeloid	ITGB2
0	1.36511	0.654	0.122	0	Myeloid	IFNGR2
0	1.360354	0.771	0.22	0	Myeloid	LYN
0	1.359446	0.588	0.146	0	Myeloid	IFI44L
0	1.354926	0.646	0.125	0	Myeloid	CASP1
0	1.354731	0.89	0.515	0	Myeloid	S100A10
0	1.350525	0.598	0.102	0	Myeloid	AGTRAP
0	1.342531	0.574	0.101	0	Myeloid	NCF1
0	1.332364	0.505	0.039	0	Myeloid	CD86
0	1.325663	0.635	0.16	0	Myeloid	OAS1
0	1.325609	0.676	0.159	0	Myeloid	SDCBP
0	1.324542	0.583	0.089	0	Myeloid	CAPG
0	1.320473	0.684	0.161	0	Myeloid	TNFRSF1B
0	1.310436	0.655	0.161	0	Myeloid	ZFAND5
0	1.309268	0.513	0.044	0	Myeloid	C19orf38
0	1.30719	0.621	0.106	0	Myeloid	THEMIS2
0	1.291408	0.547	0.097	0	Myeloid	JAK2
0	1.289703	0.577	0.104	0	Myeloid	CTSA
0	1.271551	0.577	0.181	0	Myeloid	VAMP5
0	1.262876	0.635	0.155	0	Myeloid	FBXL5
0	1.255909	0.563	0.121	0	Myeloid	IFI44
0	1.25559	0.525	0.057	0	Myeloid	C9orf72
0	1.25548	0.556	0.092	0	Myeloid	NUP214
0	1.250307	0.733	0.225	0	Myeloid	GLIPR1
0	1.247795	0.596	0.099	0	Myeloid	SCPEP1
0	1.241532	0.58	0.1	0	Myeloid	GRINA
0	1.241005	0.825	0.35	0	Myeloid	SAMHD1
0	1.23199	0.623	0.125	0	Myeloid	ADA2
0	1.22062	0.592	0.112	0	Myeloid	IFNGR1
0	1.198799	0.54	0.097	0	Myeloid	BACH1
0	1.198444	0.621	0.131	0	Myeloid	AOAH
0	1.195164	0.568	0.1	0	Myeloid	IL17RA
0	1.181906	0.712	0.225	0	Myeloid	ATP6V0B
0	1.181763	0.581	0.097	0	Myeloid	CTSH
0	1.174285	0.549	0.123	0	Myeloid	MX2
0	1.165078	0.517	0.067	0	Myeloid	ATP6V1B2
0	1.157469	0.691	0.204	0	Myeloid	CD63
0	1.156023	0.543	0.09	0	Myeloid	OGFRL1
0	1.150013	0.723	0.266	0	Myeloid	GLRX
0	1.147325	0.611	0.184	0	Myeloid	EPSTI1
0	1.140911	0.732	0.211	0	Myeloid	MYO1F
0	1.135837	0.591	0.131	0	Myeloid	PPT1
0	1.116733	0.635	0.152	0	Myeloid	PLEKHO1

0	1.116254	0.573	0.199	0	Myeloid	MX1
0	1.115878	0.796	0.366	0	Myeloid	GSTP1
0	1.112301	0.583	0.181	0	Myeloid	IRF7
0	1.104656	0.654	0.197	0	Myeloid	GSTO1
0	1.092474	0.536	0.202	0	Myeloid	GBP1
0	1.085501	0.696	0.228	0	Myeloid	LYST
0	1.084946	0.581	0.128	0	Myeloid	PECAM1
0	1.08129	0.684	0.243	0	Myeloid	SERPINB1
0	1.07782	0.69	0.215	0	Myeloid	MACROH2A1
0	1.075204	0.591	0.139	0	Myeloid	PPP1R15A
0	1.074659	0.769	0.341	0	Myeloid	PKM
0	1.069377	0.621	0.139	0	Myeloid	MARCHF1
0	1.06447	0.563	0.144	0	Myeloid	NFKBIZ
0	1.063301	0.537	0.113	0	Myeloid	RTN3
0	1.049932	0.537	0.11	0	Myeloid	GLIPR2
0	1.045498	0.623	0.183	0	Myeloid	DYNLT1
0	1.044352	0.816	0.385	0	Myeloid	KLF6
0	1.03906	0.673	0.222	0	Myeloid	WSB1
0	1.034559	0.582	0.114	0	Myeloid	ALOX5
0	1.034242	0.679	0.259	0	Myeloid	RNF149
0	1.028279	0.817	0.425	0	Myeloid	MCL1
0	1.022314	0.719	0.243	0	Myeloid	ARRB2
0	1.010129	0.777	0.521	0	Myeloid	LY6E
0	1.008831	0.502	0.099	0	Myeloid	RILPL2
0	0.989983	0.522	0.102	0	Myeloid	SKAP2
0	0.985282	0.704	0.28	0	Myeloid	PRELID1
0	0.981683	0.739	0.287	0	Myeloid	RAC1
0	0.978666	0.566	0.123	0	Myeloid	LY86
0	0.96875	0.707	0.269	0	Myeloid	RHOG
0	0.96407	0.611	0.184	0	Myeloid	PICALM
0	0.963596	0.941	0.713	0	Myeloid	GAPDH
0	0.95655	0.738	0.316	0	Myeloid	USP15
0	0.95191	0.771	0.374	0	Myeloid	EVI2B
0	0.947843	0.523	0.099	0	Myeloid	SCIMP
0	0.931808	0.782	0.35	0	Myeloid	CAST
0	0.931075	0.603	0.228	0	Myeloid	TUBA1A
0	0.924137	0.529	0.129	0	Myeloid	SNX10
0	0.921621	0.538	0.133	0	Myeloid	ATP6V0D1
0	0.920869	0.606	0.181	0	Myeloid	RNH1
0	0.916478	0.755	0.381	0	Myeloid	XAF1
0	0.916299	0.57	0.156	0	Myeloid	EHBP1L1
0	0.911784	0.548	0.138	0	Myeloid	MYD88
0	0.905858	0.545	0.151	0	Myeloid	HSBP1
0	0.895719	0.599	0.149	0	Myeloid	SYK

0	0.887236	0.551	0.154	0	Myeloid	ATG3
0	0.886818	0.564	0.172	0	Myeloid	NCOA4
0	0.885364	0.542	0.176	0	Myeloid	METTL9
0	0.884725	0.96	0.807	0	Myeloid	SH3BGRL3
0	0.878651	0.55	0.199	0	Myeloid	PARP14
0	0.874321	0.713	0.449	0	Myeloid	PLAC8
0	0.859651	0.892	0.581	0	Myeloid	JUNB
0	0.855013	0.855	0.515	0	Myeloid	GNAI2
0	0.849893	0.758	0.433	0	Myeloid	IER2
0	0.844053	0.933	0.699	0	Myeloid	CYBA
0	0.834326	0.749	0.262	0	Myeloid	EFHD2
0	0.832292	0.569	0.186	0	Myeloid	RAB10
0	0.827588	0.624	0.221	0	Myeloid	AP2S1
0	0.822077	0.519	0.152	0	Myeloid	STAT2
0	0.819519	0.691	0.302	0	Myeloid	CSTB
0	0.805147	0.886	0.563	0	Myeloid	LCP1
0	0.790705	0.535	0.177	0	Myeloid	GPCPD1
0	0.78786	0.607	0.207	0	Myeloid	CNPY3
0	0.784862	0.6	0.22	0	Myeloid	BST2
0	0.774842	0.624	0.226	0	Myeloid	RTN4
0	0.769181	0.564	0.18	0	Myeloid	LAPTM4A
0	0.768672	0.564	0.183	0	Myeloid	MGAT1
0	0.760976	0.57	0.238	0	Myeloid	GBP2
0	0.754145	0.612	0.236	0	Myeloid	PLP2
0	0.747729	0.939	0.554	0	Myeloid	HLA-DRA
0	0.743251	0.686	0.293	0	Myeloid	CAPNS1
0	0.733683	0.638	0.273	0	Myeloid	ADGRE5
0	0.731391	0.889	0.659	0	Myeloid	ITM2B
0	0.729542	0.653	0.323	0	Myeloid	UBE2L6
0	0.72164	0.633	0.254	0	Myeloid	TXN
0	0.716501	0.538	0.18	0	Myeloid	POU2F2
0	0.716076	0.509	0.174	0	Myeloid	PARP9
0	0.705168	0.647	0.328	0	Myeloid	NEAT1
0	0.705079	0.659	0.248	0	Myeloid	PLEK
0	0.700376	0.911	0.513	0	Myeloid	HLA-DRB1
0	0.697103	0.504	0.172	0	Myeloid	TUT7
0	0.693643	0.658	0.3	0	Myeloid	VAMP8
0	0.688379	0.524	0.181	0	Myeloid	CAPZA2
0	0.676791	0.7	0.386	0	Myeloid	CCNL1
0	0.670617	0.709	0.328	0	Myeloid	IQGAP1
0	0.66924	0.78	0.406	0	Myeloid	AHNAK
0	0.667192	0.631	0.273	0	Myeloid	LAMTOR1
0	0.660601	0.65	0.274	0	Myeloid	WAS
0	0.657492	0.769	0.454	0	Myeloid	PSME2

0	0.649912	0.752	0.411	0	Myeloid	ATP5MPL
0	0.644632	0.531	0.18	0	Myeloid	VASP
0	0.640533	0.542	0.2	0	Myeloid	FERMT3
0	0.640009	0.687	0.315	0	Myeloid	GRB2
0	0.63739	0.698	0.337	0	Myeloid	SH3BGRL
0	0.634622	0.67	0.328	0	Myeloid	STAT1
0	0.633859	0.842	0.491	0	Myeloid	CLIC1
0	0.629669	0.905	0.71	0	Myeloid	IFITM2
0	0.619184	0.607	0.288	0	Myeloid	DOCK8
0	0.615836	0.556	0.192	0	Myeloid	PARVG
0	0.611387	0.514	0.188	0	Myeloid	MIS18BP1
0	0.602604	0.986	0.938	0	Myeloid	HLA-B
0	0.594915	0.711	0.366	0	Myeloid	SERP1
0	0.594482	0.831	0.554	0	Myeloid	RHOA
0	0.594112	0.785	0.454	0	Myeloid	AC010894.3
0	0.588638	0.983	0.973	0	Myeloid	MT-ND1
0	0.582244	0.606	0.268	0	Myeloid	ATP6V1F
0	0.577642	0.774	0.426	0	Myeloid	LRRFIP1
0	0.577277	0.557	0.213	0	Myeloid	RGS19
0	0.571893	0.61	0.285	0	Myeloid	CD55
0	0.5691	0.507	0.183	0	Myeloid	HEBP2
0	0.566888	0.662	0.315	0	Myeloid	GNB2
0	0.563931	0.775	0.474	0	Myeloid	ATP6V0E1
0	0.561719	0.6	0.296	0	Myeloid	IRF1
0	0.53854	0.528	0.213	0	Myeloid	ATP6AP2
0	0.537732	0.733	0.419	0	Myeloid	COX5B
0	0.529718	0.759	0.447	0	Myeloid	ARPC5
0	0.525511	0.928	0.805	0	Myeloid	SERF2
0	0.524	0.622	0.302	0	Myeloid	NOP10
0	0.521267	0.639	0.301	0	Myeloid	NDUFB1
0	0.516572	0.685	0.366	0	Myeloid	CD44
0	0.514256	0.517	0.206	0	Myeloid	CLTA
0	0.510155	0.73	0.406	0	Myeloid	TPI1
0	0.503759	0.67	0.334	0	Myeloid	PSMB3
0	0.502468	0.742	0.423	0	Myeloid	ACTR2
0	0.502017	0.603	0.277	0	Myeloid	RAB7A
0	0.500897	0.507	0.198	0	Myeloid	TMEM219
0	3.092236	0.728	0.115	0	T	IL32
0	3.004172	0.605	0.098	0	T	TRBC1
0	2.928282	0.687	0.089	0	T	CD3D
0	2.917116	0.699	0.116	0	T	TRAC
0	2.865163	0.569	0.073	0	T	IL7R
0	2.439905	0.64	0.068	0	T	CD3E
0	2.381506	0.787	0.324	0	T	TRBC2

0	2.11567	0.532	0.062	0	T	CD7
0	1.935681	0.543	0.068	0	T	LCK
0	1.766938	0.591	0.176	0	T	PCED1B-AS1
0	1.687017	0.666	0.34	0	T	AC010197.2
0	1.675913	0.533	0.155	0	T	ARL4C
0	1.663299	0.795	0.453	0	T	GIMAP7
0	1.544785	0.649	0.219	0	T	ETS1
0	1.365213	0.687	0.322	0	T	EVL
0	1.26049	0.534	0.282	0	T	RBL2
0	1.152623	0.645	0.477	0	T	HCST
0	1.06414	0.81	0.693	0	T	CALM1
0	0.973952	0.745	0.516	0	T	TLE5
0	0.848257	0.604	0.43	0	T	GIMAP4
0	0.847415	0.706	0.6	0	T	SARAF
0	0.846466	0.949	0.933	0	T	RPS3
0	0.844846	0.645	0.497	0	T	TRAF3IP3
0	0.810693	0.917	0.913	0	T	RPS12
0	0.781985	0.675	0.553	0	T	NPM1
0	0.76586	0.703	0.586	0	T	HSPA8
0	0.752416	0.512	0.417	0	T	CCND3
0	0.744746	0.999	0.999	0	T	MALAT1
0	0.703875	0.586	0.46	0	T	FAM172A
0	0.702054	0.974	0.972	0	T	RPS29
0	0.686077	0.951	0.938	0	T	RPS6
0	0.677052	0.922	0.901	0	T	RPL14
0	0.671728	0.83	0.789	0	T	RPL3
0	0.660398	0.614	0.519	0	T	JAK1
0	0.651271	0.943	0.937	0	T	RPS27A
0	0.645033	0.817	0.794	0	T	RPS15A
0	0.639016	0.936	0.946	0	T	PFN1
0	0.638308	0.509	0.313	0	T	CLEC2D
0	0.632762	0.978	0.973	0	T	RPL13
0	0.630994	0.844	0.82	0	T	RPS4X
0	0.625538	0.722	0.543	0	T	FYB1
0	0.623701	0.876	0.869	0	T	RPS3A
0	0.609697	0.951	0.952	0	T	RPL32
0	0.607937	0.721	0.648	0	T	RPSA
0	0.607287	0.708	0.677	0	T	RAC2
0	0.593296	0.967	0.966	0	T	RPL31
0	0.591072	0.785	0.763	0	T	AL136454.1
0	0.585015	0.945	0.946	0	T	RPS18
0	0.578718	0.944	0.952	0	T	RPS14
0	0.575204	0.972	0.974	0	T	RPLP2
0	0.573584	0.728	0.7	0	T	EEF1B2

0	0.573447	0.946	0.935	0	T	RPL13A
0	0.573123	0.846	0.85	0	T	RPS25
0	0.56742	0.938	0.939	0	T	RPL5
0	0.55997	0.674	0.652	0	T	BAIAP2L1
0	0.557908	0.932	0.922	0	T	AC099560.1
0	0.554449	0.933	0.933	0	T	RPL10
0	0.554346	0.948	0.952	0	T	RPL30
0	0.552782	0.787	0.742	0	T	MYL12A
0	0.547639	0.966	0.961	0	T	RPS27
0	0.543551	0.92	0.924	0	T	RPS16
0	0.536484	0.907	0.907	0	T	RPS20
0	0.530553	0.931	0.928	0	T	RPL36
0	0.528318	0.631	0.58	0	T	NCL
0	0.521865	0.954	0.961	0	T	RPL34
0	0.519618	0.843	0.845	0	T	AMBRA1
0	0.501087	0.741	0.722	0	T	RPL10A
4.33E-279	0.589983	0.51	0.445	1.14E-274	T	BIN2
3.72E-277	0.535694	0.804	0.793	9.83E-273	T	ACTG1
2.82E-255	0.535532	0.549	0.504	7.45E-251	T	SNRPD2
0	4.489025	0.878	0.069	0	B	MS4A1
0	4.203548	0.942	0.44	0	B	IGHM
0	3.973969	0.669	0.37	0	B	IGLC2
0	3.755303	0.734	0.044	0	B	IGHD
0	3.534371	0.84	0.052	0	B	CD79A
0	3.516912	0.607	0.029	0	B	TCL1A
0	3.051388	0.698	0.046	0	B	BANK1
0	2.792491	0.605	0.025	0	B	LINC00926
0	2.493442	0.544	0.023	0	B	FCRL1
0	2.384827	0.987	0.846	0	B	CD74
0	2.339728	0.51	0.021	0	B	CD22
0	2.261537	0.544	0.051	0	B	RALGPS2
0	2.137507	0.693	0.171	0	B	HLA-DQA1
0	2.019092	0.557	0.171	0	B	HVCN1
0	1.919942	0.576	0.189	0	B	MEF2C
0	1.903388	0.855	0.457	0	B	HLA-DPB1
0	1.895555	0.88	0.604	0	B	CD37
0	1.805036	0.942	0.592	0	B	HLA-DRA
0	1.718212	0.67	0.279	0	B	HLA-DQB1
0	1.704855	0.51	0.12	0	B	HLA-DQA2
0	1.674131	0.785	0.375	0	B	FCMR
0	1.661989	0.626	0.263	0	B	HLA-DMB
0	1.629913	0.849	0.459	0	B	HLA-DPA1
0	1.622495	0.92	0.55	0	B	HLA-DRB1
0	1.264841	0.762	0.422	0	B	LTB

0	1.221303	0.543	0.311	0	B	SNX2
0	1.107271	0.538	0.321	0	B	CXCR4
0	0.923307	0.933	0.817	0	B	RPL18A
0	0.91758	0.547	0.359	0	B	EZR
0	0.871457	0.58	0.41	0	B	ISG20
0	0.808991	0.97	0.907	0	B	TXNRD1
0	0.744277	0.836	0.762	0	B	BTG1
0	0.734609	0.915	0.777	0	B	RPS5
0	0.705822	0.977	0.926	0	B	CD52
0	0.665268	0.987	0.957	0	B	RPS27
0	0.66406	0.958	0.893	0	B	RPL8
0	0.649482	0.954	0.902	0	B	RPS11
0	0.589014	0.976	0.918	0	B	RPL23A
0	0.583162	0.968	0.915	0	B	AC099560.1
0	0.577726	0.978	0.935	0	B	RPL15
0	0.561216	0.968	0.907	0	B	RPS23
0	0.525084	0.929	0.843	0	B	RPLP0
0	0.521338	0.974	0.931	0	B	RPL13A
0	0.502213	0.915	0.86	0	B	RPL12
3.17E-306	0.592874	0.529	0.375	8.38E-302	B	CLEC2D
9.60E-293	0.731863	0.514	0.41	2.54E-288	B	SNX3
3.03E-287	0.615988	0.63	0.535	8.00E-283	B	UCP2
1.37E-272	0.548549	0.769	0.705	3.62E-268	B	LAPTM5
1.38E-271	0.660998	0.538	0.436	3.65E-267	B	ZFP36L1
6.66E-247	0.568608	0.508	0.384	1.76E-242	B	CD81
9.95E-186	0.519159	0.543	0.462	2.63E-181	B	CIRBP
4.65E-117	0.510891	0.533	0.493	1.23E-112	B	JUND
0	3.37554	0.834	0.037	0	NK	XCL2
0	3.106769	0.789	0.019	0	NK	XCL1
0	2.899768	0.899	0.174	0	NK	CTSW
0	2.657576	0.701	0.051	0	NK	KLRF1
0	2.571003	0.834	0.141	0	NK	KLRD1
0	2.494728	0.586	0.037	0	NK	KLRC1
0	2.409221	0.682	0.091	0	NK	IL2RB
0	2.157253	0.699	0.078	0	NK	TRDC
2.03E-280	3.446904	0.918	0.239	5.36E-276	NK	GNLY
1.53E-249	2.077545	0.639	0.1	4.05E-245	NK	KLRB1
1.47E-222	2.514046	0.715	0.158	3.89E-218	NK	CMC1
7.57E-215	2.100119	0.676	0.132	2.00E-210	NK	HOPX
5.41E-202	1.880636	0.541	0.086	1.43E-197	NK	GZMK
1.28E-200	2.373352	0.887	0.287	3.38E-196	NK	CD7
3.79E-164	1.774746	0.944	0.338	1.00E-159	NK	NKG7
2.31E-139	1.477665	0.749	0.209	6.10E-135	NK	PRF1
1.52E-134	1.642061	0.969	0.766	4.03E-130	NK	IFITM2

8.09E-116	1.898821	0.834	0.394	2.14E-111	NK	ID2
1.10E-107	1.104794	0.727	0.22	2.91E-103	NK	CST7
1.25E-101	1.812191	0.555	0.166	3.30E-97	NK	DUSP2
3.74E-78	1.403821	0.634	0.252	9.88E-74	NK	RUNX3
8.06E-78	1.560154	0.651	0.263	2.13E-73	NK	CD2
3.92E-67	1.487508	0.538	0.186	1.04E-62	NK	GZMB
5.13E-63	1.286463	0.577	0.218	1.36E-58	NK	GZMA
1.83E-57	1.15457	0.834	0.557	4.84E-53	NK	HCST
1.10E-50	1.157938	0.792	0.564	2.90E-46	NK	JAK1
1.78E-50	0.618686	0.992	0.941	4.70E-46	NK	PFN1
6.58E-49	1.03315	0.673	0.336	1.74E-44	NK	ARL4C
4.06E-48	0.75665	0.834	0.41	1.07E-43	NK	FCER1G
5.22E-45	0.582316	0.93	0.486	1.38E-40	NK	TYROBP
1.50E-44	0.934219	0.882	0.6	3.97E-40	NK	SRGN
1.36E-39	1.011693	0.617	0.345	3.59E-35	NK	CD63
2.41E-39	1.021607	0.623	0.334	6.37E-35	NK	STK17A
1.07E-36	0.968165	0.569	0.298	2.83E-32	NK	SLFN5
2.17E-34	1.019132	0.775	0.619	5.74E-30	NK	SELL
3.41E-33	0.872772	0.766	0.525	9.02E-29	NK	PLAC8
1.50E-31	0.694002	0.899	0.789	3.97E-27	NK	UBB
9.23E-29	0.907606	0.507	0.283	2.44E-24	NK	XBP1
1.63E-27	0.697922	0.879	0.777	4.31E-23	NK	BTG1
4.87E-27	0.605904	0.89	0.749	1.29E-22	NK	CALM1
1.15E-26	0.654656	0.569	0.31	3.03E-22	NK	CCL5
8.16E-25	0.839584	0.589	0.398	2.16E-20	NK	PRMT2
1.44E-24	0.685361	0.51	0.274	3.80E-20	NK	CD69
4.18E-24	0.71303	0.8	0.595	1.10E-19	NK	LY6E
5.87E-23	0.738549	0.758	0.615	1.55E-18	NK	ZFP36L2
5.53E-21	0.706114	0.693	0.53	1.46E-16	NK	SEPTIN7
9.62E-20	0.745171	0.87	0.81	2.54E-15	NK	TXNIP
1.05E-19	0.761517	0.597	0.404	2.78E-15	NK	EFHD2
3.68E-19	0.604474	0.789	0.692	9.73E-15	NK	RAC2
8.23E-18	0.60615	0.513	0.315	2.17E-13	NK	SLC9A3R1
1.23E-16	0.817642	0.535	0.388	3.24E-12	NK	CFLAR
3.78E-16	0.603182	0.727	0.625	9.98E-12	NK	FXYD5
1.35E-15	0.567507	0.749	0.593	3.57E-11	NK	CLIC1
6.44E-15	0.602516	0.53	0.358	1.70E-10	NK	FYN
2.60E-14	0.696573	0.546	0.393	6.88E-10	NK	PLAAT4
2.72E-14	0.599111	0.538	0.377	7.19E-10	NK	MACF1
6.53E-14	0.515939	0.673	0.498	1.72E-09	NK	EVL
3.19E-13	0.553829	0.744	0.628	8.43E-09	NK	TAGLN2
3.70E-13	0.630225	0.594	0.446	9.77E-09	NK	ISG20
3.77E-13	0.589713	0.634	0.5	9.97E-09	NK	TPI1
2.78E-12	0.535005	0.527	0.379	7.35E-08	NK	ADGRE5

3.15E-12	0.582203	0.592	0.476	8.33E-08	NK	BIN2
4.06E-11	0.525926	0.656	0.528	1.07E-06	NK	IER2
1.49E-10	0.592101	0.518	0.401	3.93E-06	NK	SERBP1
1.09E-09	0.523511	0.62	0.491	2.87E-05	NK	GSTP1
4.57E-09	0.624245	0.532	0.437	0.000121	NK	TGFB1
5.39E-07	0.549682	0.6	0.543	0.014243	NK	TSC22D3

Table S2. Lists of differentially expressed genes for each group (Normal, GMPP, RMPP) relative to the other two groups

p_val	avg_log2FC	pct.1	pct.2	p_val_adj	group	gene
0	1.60793611	0.611	0.33	0	RMPP	IGLC2
0	1.236710705	0.76	0.422	0	RMPP	IGHM
0	0.789125686	0.602	0.437	0	RMPP	HSP90B1
0	0.66971811	0.507	0.356	0	RMPP	IL32
0	0.617423803	0.853	0.766	0	RMPP	ACTG1
0	0.604036321	0.521	0.363	0	RMPP	NEAT1
0	0.600470254	0.69	0.55	0	RMPP	SUB1
0	0.592579972	0.849	0.739	0	RMPP	GAPDH
2.84E-231	0.52896349	0.585	0.458	7.49E-227	RMPP	PPIB
2.68E-100	0.502116198	0.545	0.449	7.09E-96	RMPP	TUBA1B
0	1.701720937	0.504	0.096	0	Normal	RPS4Y1
0	1.054500804	0.625	0.378	0	Normal	FCMR
0	1.026053124	0.69	0.392	0	Normal	LTB
0	0.743591893	0.882	0.821	0	Normal	RPL18A
0	0.720073008	0.689	0.65	0	Normal	CD37
0	0.714735193	0.738	0.623	0	Normal	BAIAP2L1
0	0.693136003	0.868	0.778	0	Normal	RPL3
0	0.689625778	0.553	0.474	0	Normal	JUND
0	0.661436684	0.824	0.747	0	Normal	AL136454.1
0	0.659027217	0.918	0.848	0	Normal	RPS3A
0	0.657581461	0.966	0.927	0	Normal	RPL13A
0	0.652640204	0.952	0.904	0	Normal	RPS23
0	0.637312545	0.99	0.964	0	Normal	RPLP2
0	0.627810555	0.915	0.849	0	Normal	RPL12
0	0.626429542	0.987	0.962	0	Normal	RPL37
0	0.613959943	0.975	0.939	0	Normal	RPL32
0	0.597667296	1	0.999	0	Normal	MALAT1
0	0.589034736	0.753	0.681	0	Normal	PNRC1
0	0.587995934	0.856	0.781	0	Normal	RPS5
0	0.587179012	0.96	0.919	0	Normal	RPL10
0	0.585316465	0.768	0.685	0	Normal	EEF1B2
0	0.580711029	0.981	0.954	0	Normal	RPS27
0	0.578566532	0.749	0.692	0	Normal	SRSF5
0	0.571919119	0.689	0.625	0	Normal	NBEAL1
0	0.569836435	0.961	0.929	0	Normal	RPS27A
0	0.560915382	0.957	0.916	0	Normal	RPL23A
0	0.553702086	0.945	0.899	0	Normal	RPS12
0	0.553604277	0.969	0.931	0	Normal	RPS6
0	0.550419414	0.939	0.911	0	Normal	TXNRD1
0	0.548465117	0.888	0.827	0	Normal	RPS25
0	0.546250868	0.971	0.94	0	Normal	RPL30

0	0.545686471	0.68	0.6	0	Normal	TLE5
0	0.538291468	0.966	0.935	0	Normal	RPS18
0	0.534312156	0.978	0.947	0	Normal	RPL34
0	0.533504942	0.646	0.497	0	Normal	TRBC2
0	0.522320226	0.937	0.89	0	Normal	RPS8
0	0.516954914	0.965	0.939	0	Normal	RPS14
0	0.509644319	0.959	0.929	0	Normal	RPS13
0	0.507464913	0.984	0.957	0	Normal	RPL31
0	0.500211596	0.987	0.969	0	Normal	RPL13
2.53E-280	0.587946292	0.529	0.453	6.68E-276	Normal	CIRBP
4.87E-254	0.50501552	0.533	0.422	1.29E-249	Normal	JUN
1.33E-239	0.857921026	0.884	0.872	3.51E-235	Normal	CD74
2.22E-226	0.506980547	0.601	0.552	5.86E-222	Normal	TRAF3IP3
1.71E-100	0.516067431	0.624	0.633	4.53E-96	Normal	HLA-DRB1
1.11E-41	0.521663858	0.528	0.551	2.94E-37	Normal	HLA-DPB1
0	2.426415175	0.818	0.416	0	GMPP	IFITM3
0	2.212302698	0.696	0.209	0	GMPP	IFI6
0	2.131334998	0.663	0.123	0	GMPP	IFI44L
0	2.119288728	0.73	0.276	0	GMPP	ISG15
0	1.745294517	0.817	0.363	0	GMPP	XAF1
0	1.654598523	0.62	0.186	0	GMPP	MX1
0	1.631121397	0.658	0.308	0	GMPP	MT2A
0	1.570053415	0.827	0.505	0	GMPP	LY6E
0	1.56362025	0.792	0.427	0	GMPP	LYZ
0	1.516624868	0.612	0.297	0	GMPP	TNFSF10
0	1.499726262	0.544	0.221	0	GMPP	SERPINA1
0	1.488455909	0.612	0.311	0	GMPP	AIF1
0	1.442792808	0.746	0.388	0	GMPP	TYROBP
0	1.432451933	0.632	0.301	0	GMPP	FCN1
0	1.430670568	0.667	0.364	0	GMPP	CST3
0	1.384730735	0.651	0.319	0	GMPP	FCER1G
0	1.380104186	0.558	0.198	0	GMPP	OAS1
0	1.35095718	0.764	0.522	0	GMPP	CTSS
0	1.337289244	0.569	0.193	0	GMPP	IRF7
0	1.325711104	0.738	0.495	0	GMPP	PSAP
0	1.315075737	0.512	0.148	0	GMPP	IFI44
0	1.297890113	0.51	0.215	0	GMPP	FGL2
0	1.283910037	0.576	0.204	0	GMPP	EPSTI1
0	1.261491672	0.533	0.245	0	GMPP	LST1
0	1.180235481	0.56	0.289	0	GMPP	GRN
0	1.13423673	0.749	0.433	0	GMPP	FOS
0	1.121903954	0.513	0.293	0	GMPP	HLA-DRB5
0	1.104829507	0.977	0.922	0	GMPP	FTH1
0	1.096776685	0.724	0.473	0	GMPP	SAT1

0	1.09535021	0.564	0.267	0	GMPP	MNDA
0	1.084766131	0.746	0.479	0	GMPP	S100A11
0	1.03459347	0.511	0.254	0	GMPP	CD14
0	1.00748004	0.975	0.943	0	GMPP	FTL
0	0.984512242	0.534	0.303	0	GMPP	NPC2
0	0.933766408	0.703	0.441	0	GMPP	DUSP1
0	0.899719728	0.824	0.515	0	GMPP	S100A9
0	0.881032259	0.564	0.342	0	GMPP	BRI3
0	0.875158334	0.787	0.529	0	GMPP	SRGN
0	0.87093135	0.613	0.345	0	GMPP	UBE2L6
0	0.850880309	0.527	0.292	0	GMPP	CEBPB
0	0.841666044	0.663	0.378	0	GMPP	TRIM22
0	0.839953568	0.883	0.722	0	GMPP	IFITM2
0	0.831589398	0.524	0.256	0	GMPP	BST2
0	0.822877171	0.545	0.274	0	GMPP	SAMD9L
0	0.815657573	0.844	0.666	0	GMPP	S100A6
0	0.808161334	0.575	0.361	0	GMPP	C1orf162
0	0.798321735	0.779	0.493	0	GMPP	S100A8
0	0.79531885	0.635	0.347	0	GMPP	STAT1
0	0.77526955	0.847	0.672	0	GMPP	S100A4
0	0.749587393	0.541	0.328	0	GMPP	CARD16
0	0.748030306	0.603	0.386	0	GMPP	VSIR
0	0.712849831	0.595	0.403	0	GMPP	ANXA2
0	0.710052275	0.738	0.563	0	GMPP	ITGB2
0	0.706095534	0.987	0.938	0	GMPP	HLA-B
0	0.687626697	0.616	0.435	0	GMPP	NFKBIA
0	0.683679981	0.667	0.471	0	GMPP	PLAC8
0	0.651794119	0.692	0.493	0	GMPP	ZFP36
0	0.6360864	0.654	0.424	0	GMPP	SAMHD1
0	0.626835884	0.692	0.49	0	GMPP	PSME2
0	0.623521783	0.501	0.302	0	GMPP	FGR
0	0.620125446	0.5	0.316	0	GMPP	PYCARD
0	0.609311166	0.539	0.346	0	GMPP	GLRX
0	0.584232422	0.716	0.508	0	GMPP	RNF213
0	0.578485488	0.643	0.498	0	GMPP	COTL1
0	0.561086886	0.501	0.335	0	GMPP	LYN
0	0.545869684	0.959	0.886	0	GMPP	HLA-C
0	0.531236989	0.562	0.372	0	GMPP	TKT
0	0.512072709	0.653	0.463	0	GMPP	IFI16
0	0.504066953	0.594	0.392	0	GMPP	LGALS1
